# Theoretical assessment of persistence and adaptation in weeds with complex life cycles

**DOI:** 10.1038/s41477-023-01482-1

**Published:** 2023-08-03

**Authors:** Dana Lauenroth, Chaitanya S. Gokhale

**Affiliations:** 1 Research Group for Theoretical Models of Eco-evolutionary Dynamics, Department Theoretical Biology, Max Planck Institute for Evolutionary Biology, Plön, Germany; 2 Center for Computational and Theoretical Biology, Julius-Maximilians-Universität Würzburg, Würzburg, Germany

**Keywords:** Plant ecology, Theoretical ecology, Population dynamics

## Abstract

Herbicide-resistant weeds pose a substantial threat to global food security. Perennial weed species are particularly troublesome. Such perennials as *Sorghum halepense* spread quickly and are difficult to manage due to their ability to reproduce sexually via seeds and asexually through rhizomes. Our theoretical study of *S. halepense* incorporates this complex life cycle with control measures of herbicide application and tillage. Rooted in the biology and experimental data of *S. halepense*, our population-based model predicts population dynamics and target-site resistance evolution in this perennial weed. We found that the resistance cost determines the standing genetic variation for herbicide resistance. The sexual phase of the life cycle, including self-pollination and seed bank dynamics, contributes substantially to the persistence and rapid adaptation of *S. halepense*. While self-pollination accelerates target-site resistance evolution, seed banks considerably increase the probability of escape from control strategies and maintain genetic variation. Combining tillage and herbicide application effectively reduces weed densities and the risk of control failure without delaying resistance adaptation. We also show how mixtures of different herbicide classes are superior to rotations and mono-treatment in controlling perennial weeds and resistance evolution. Thus, by integrating experimental data and agronomic views, our theoretical study synergistically contributes to understanding and tackling the global threat to food security from resistant weeds.

## Abstract (web summary)

The perennial weed, Johnsongrass, is a threat to global food security. Its two reproduction modes drive acquisition of herbicide resistance, but a well-strategized blend of tillage and diverse herbicides offers effective countermeasures.

## Main

*Sorghum halepense* (Johnsongrass) is infamous for being “a threat to native grasslands and agriculture”^1^ (p. 413). Johnsongrass is classified as a weed in 53 countries^1^ and was ranked as the world’s sixth-worst weed in 1977^2^. It causes considerable yield losses in 30 different crops, including essential cash crops such as corn, cotton, sugarcane and soybean^1^. The grassy weed out-competes native grasses like big bluestem (*Andropogon gerardii*), little bluestem (*Schizachyrium scoparius*) and switchgrass (*Panicum virgatum*) in grasslands^3^. Johnsongrass is a highly competitive and invasive perennial, capable of sexual reproduction via seeds and asexual propagation through rhizomes^3–5^. In addition to resource competition, Johnsongrass affects crop production by serving as an alternative host for various agricultural pests^1,5^.

In cropped areas, typical control of Johnsongrass is by a postemergence application of herbicides, particularly glyphosate, acetyl-coenzyme-A carboxylase (ACCase)-inhibitor and acetolactate synthase (ALS)-inhibitor^1,5^. However, the continuous use of such herbicides has caused Johnsongrass to develop resistances, including target-site resistance towards various ACCase- and ALS-inhibitors^5,6^ and non-target-site resistance to glyphosate^7^. Herbicide resistance imposes a considerable challenge on weed management. There is an urgent need for weed management strategies that reduce herbicide resistance risk while effectively controlling the weed population. Integrated approaches, combining herbicide application with mechanical measures, such as tillage and potentially crop rotations, are reported to provide efficient control of Johnsongrass, including its rhizomes^4,5^. Mixtures and rotations of herbicides with different sites of action reportedly reduce weed densities more efficiently than recurrent use of one herbicide class and delay resistance evolution^8–10^.

To develop and select sustainable control strategies, we must improve our understanding of eco-evolutionary processes underlying the population dynamics and how inherent plant characteristics shape the evolution of herbicide resistance in perennial weeds. Mathematical modelling has proven valuable in studying long-term population dynamics and herbicide resistance evolution in weeds^11,12^. However, most existing models deal with annual weeds; the complex life cycle of perennial species has been addressed to a lesser extent^11^ (but see refs. ^13,14^). To our knowledge, only two population dynamical models exist for Johnsongrass and more general for perennials comprising the whole life cycle^13,14^. The earlier study presented an individual-based model, which captures self-pollination but lacks a seed bank^13^. A recent stage-structured model comprises both a seed bank and a bank of rhizome buds^14^. However, the model lacks the possibility of de novo resistance evolution and pure cross-pollination is assumed.

The goals of this study are (1) to explore how the resistance cost and its dominance affect the standing genetic variation for target-site resistance; (2) to study how genetic details, such as the dominance of resistance and the associated fitness cost, impact population dynamics and target-site resistance evolution in herbicide-treated weeds; (3) to examine the effects of self-pollination and seed bank formation on genetic diversity, target-site resistance evolution and risk of control failure in perennial species; and (4) to determine the potential of tillage, herbicide mixtures and rotations for controlling resistance evolution and weed control failure. To this aim, we implemented the life cycle of Johnsongrass, both mathematically and computationally. The sexual and asexual phases are incorporated differentially since the spread of resistance is explicitly genetic.

Even though the model is presented in the context of Johnsongrass, it can capture the population dynamics and herbicide resistance adaptation of general perennial weeds. More generally, our population-based approach can be adapted to any species that comprise sexual reproduction in the continuum of selfing and outcrossing, asexual propagation and potentially a seed bank component.

## Model summary

The seminal work by Sager and Mortimer^15^ has inspired several models of weed life cycles. The population dynamic is typically captured deterministically using several discrete time equations, each corresponding to the included life stages^11^. Often the population is assumed to be even-aged, with system time steps of one-year increments^11^. We introduce a detailed population-based model describing the dynamics of seed- and rhizome-propagated Johnsongrass. Our deterministic model with an annual time step comprises the whole perennial life cycle. We then use the model to forecast population dynamics and the evolution of target-site resistance. In this section, we summarize the main features of our theoretical model: life-history stages and ecobiology, control measures and resistance and stochastic simulations. For a detailed exposition, we refer to the Methods. We inferred all model parameters carefully from field trials in the literature. In the Supplementary Information, we provide the detailed derivation of all parameter values and a summary in Table 1.

### Life cycle

Johnsongrass is dormant throughout the winter, overwintering as seeds or rhizomes in the ground^4^. At the beginning of a growing season, seeds germinate to produce seedlings and shoots emerge from nodes on the rhizomes^5^. Mature plants produce new rhizomes and, after flowering, viable seeds^4,5^.

**Fig. 1:**
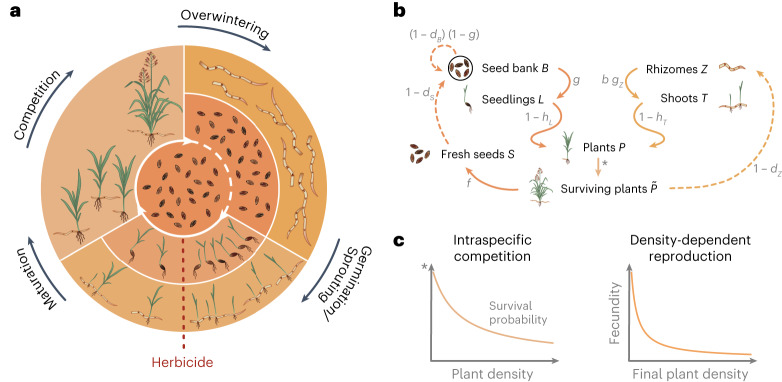
Schematic illustration of the life cycle of Johnsongrass and its representation in our model. **a**, The life cycle of Johnsongrass. Johnsongrass reproduces sexually via seeds (inner ring) and asexually through rhizomes (outer ring). Seeds can stay dormant in the ground for several years, forming a seed bank (central circle). New seeds and seeds from the seed bank might germinate in spring or stay dormant as part of the seed bank (expressed by the dotted line). Rhizomes give rise to shoots in the first spring after their production. Herbicide application (red dotted line) can kill susceptible seedlings and shoots. The plants that survive, then compete for resources as they mature. The aboveground plant material dies in winter and Johnsongrass overwinters as seeds and rhizomes in the ground. **b**, Schematic representation of our model. The left side corresponds to the sexual reproduction of Johnsongrass and the right side represents the asexual propagation. Solid arrows depict within-season dynamics and dashed arrows show dynamics between seasons. Survival probabilities and fecundity are shown in grey next to the corresponding arrows. **c**, Intraspecific competition. Intraspecific resource competition leads to self-thinning and density-dependent fecundity reduction. The left graph displays the probability of intraspecific competition survival in young plants (*P*) as a function of their density. The density-dependent reduction in fecundity, that is the number of seeds (*f*) and rhizome buds (*b*) produced by mature plants, is illustrated on the right.

Figure 1 outlines the life cycle of Johnsongrass with both reproduction pathways, sexual via seeds and asexual through rhizomes (panel a) and its representation in our model (panel b). The figure further illustrates the implementation of self-thinning and density-dependent reproduction resulting from intraspecific competition (panel c). Only rhizomes and seeds are present in early spring. Axillary buds formed on the nodes of rhizomes can develop as shoots or secondary rhizomes^16^. However, the apex of a rhizome exerts apical dominance over axillary buds, inhibiting their growth^17^. The number of shoots in any given season can be determined from the number of rhizomes, the number of nodes on rhizomes and the proportion of nodes producing shoots.

Freshly produced Johnsongrass seeds are highly dormant throughout the season^4^. They germinate in the subsequent seasons and can stay viable in the ground for several years^4,5^. New seeds enter the seed bank but are subject to postdispersal predation and might lose viability or decay until the beginning of the next season^18^. Each spring, only a certain proportion of seeds in the seed bank germinates to produce seedlings^19^. Non-germinated seeds may either be lost due to decay and viability loss or stay dormant^18^. At all times, the seed bank consists of non-germinated seeds from the previous seed bank and seeds produced in the preceding season.

Seedlings and shoots grow into adult plants. Even though rhizome shoots might emerge earlier than seedlings due to a lower temperature requirement for germination, similar growth and development were observed for Johnsongrass originating from seeds and rhizomes^20,21^. Hence, we do not distinguish the adult plants by their origin. The cycle continues as the adult plants produce new seeds and rhizomes, of which only a certain amount survive the winter.

### Intraspecific competition

Johnsongrass is more strongly affected by intraspecific competition than by competition from other perennial grasses, such as big bluestem, little bluestem and switchgrass^3^. Since the crop density is further assumed to remain constant throughout the seasons, we do not explicitly model competition from the crop and other weeds. We implement the hyperbolic self-thinning function introduced by ref. ^22^ to model early plant survival (Fig. 1c).

Moreover, intraspecific competition was shown to notably impact the formation of reproductive structures in Johnsongrass^23^. In the greenhouse experiment, high stand densities delayed or even prevented tillering and delayed the formation of rhizomes and panicles. Also, the final dry weight of reproductive structures was reduced in crowded stands^23^. Due to a lack of data, we assume that the total seed and rhizome yield per square metre approaches a constant value at moderate to high densities^24^ (the density-dependent fertility is illustrated in Fig. 1c).

### Control and resistance

Numerous control strategies exist for managing weed populations. They are typically classified according to their primary mode of action: chemical, mechanical, biological, cultural or allelopathic^5^. We focus on tillage (mechanical control) and postemergence herbicide application (chemical control).

As for target-site resistance, for example against ACCase- or ALS-inhibitors, we consider a single allele R associated with herbicide resistance^25–27^. We assume a diploid genome but the model can be extended to reflect higher ploidy levels (see ref. ^14^ for a comparison of the rate of herbicide resistance evolution in diploid and tetraploid weeds). The susceptible phenotype results from the homozygous genotype WW and genotype RR plants are resistant. The factor 0 ≤ *k*_*h*_ ≤ 1 captures the variability in the dominance of resistance such that we can account for recessivity (*k*_*h*_ = 0), partial dominance (0 < *k*_*h*_ < 1) and complete dominance (*k*_*h*_ = 1) of the resistance trait. Spontaneous mutations to the resistance allele R can occur during sexual reproduction. We do not consider back mutations as they do not change the dynamics relevantly. Johnsongrass is primarily self-pollinated, leading to higher homozygosity and reduced genetic diversity in the produced seeds compared to cross-fertilization^4^. The sexual reproduction in our model follows Mendelian inheritance and includes spontaneous mutations to the resistance allele R occurring with probability *μ* (Supplementary Information).

### Standing genetic variation

Indeed, while mutations can emerge due to the selection pressure of the herbicides, resistance may already exist in the natural variation in a population. Standing genetic variation for herbicide resistance has been found in plant populations never exposed to herbicides, among others, target-site resistance against ACCase-inhibitors^28^. Moreover, herbicide resistance adaptation may primarily proceed from standing genetic variants as the initial frequency of resistant individuals may be much higher than the spontaneous mutation rate of resistance alleles^27,28^. Thus, we also include standing genetic variation in our model and derive an analytic approximation of the expected variation (**v**) in an untreated field.

### Resistance cost

Herbicide resistance can be associated with a fitness cost^29^. In particular, a fitness cost on seed production associated with a mutation conferring resistance against ACCase-inhibitors has been reported in Johnsongrass^30^. As for the herbicide resistance, the model allows for dominance variability regarding the fitness cost, implemented by a dominance factor 0 ≤ *k*_*c*_ ≤ 1. Hence, for type RR and RS plants, a reduction in the average number of seeds produced by *c* and *k*_*c*_*c*, respectively, is considered compared to the fecundity of susceptible plants. The resistance cost is always relevant, irrespective of the control regime.

### Herbicide efficacy

In our model, herbicide resistance is complete for homozygous-resistant plants. Herbicide application can kill susceptible plants and partly resistant heterozygotes with an efficacy *h* and (1 − *k*_*h*_)*h*, respectively. Due to inadequate translocation of herbicides into the rhizomes and dormant buds, their control is only partial^17,31^. Thus, even when the herbicide kills shoots, new shoots may eventually resprout from the parent rhizome^17,31^. This resprouting reduces the herbicide efficacy on rhizome shoots compared to seedlings^32^. Johnsongrass rhizomes are cut into pieces during tillage and partly turned to the soil surface^4^. The fragmented rhizomes in shallow layers are exposed to low temperatures during winter, leading to increased mortality^4,33^. Additionally, rhizome fragmentation can maximize bud sprouting^34^, allowing for enhanced control of Johnsongrass plants originating from rhizomes by the herbicide^31,33^.

### Second herbicide

To study the effect of binary herbicide mixtures and rotations, we consider two classes of herbicides used for Johnsongrass control, ACCase- and ALS-inhibitors^5^. Due to the different sites of action, target-site resistance against herbicides of one class is uncorrelated with resistance against the other. However, in annual ryegrass (*Lolium rigidum*), multiple and cross-resistance to ACCase- and ALS-inhibitors has been reported due to a combination of target-site and non-target-site resistance^35^. Since we do not consider non-target-site resistance, we omit cross-resistance. The combined efficacy of the two herbicides applied as a mixture is calculated as *h*_mix_ = 1 − (1 − *h*_1_)(1 − *h*_2_) = *h*_1_ + *h*_2_ − *h*_1_*h*_2_, where *h*_1_ and *h*_2_ are the efficacies of the herbicides in mono-treatment^36^. Likewise, suppose the effects of the fitness costs associated with the respective target-site resistance are independent. Then the fitness cost of a plant fully resistant against both herbicides is *c*_double resistant_ = 1 − (1 − *c*_1_)(1 − *c*_2_) = *c*_1_ + *c*_2_ − *c*_1_*c*_2_. Here *c*_1_ and *c*_2_ are the fitness costs of resistance towards the individual herbicides.

### Stochastic simulations

Deterministic models capture selection as an evolutionary force, particularly for large populations. However, for small populations, strong selection may lead to the extinction of non-favoured types. Moreover, genetic drift acquires more relevance in small populations. We implemented stochastic simulations of our population-based model to account for such natural stochasticity. This approach allows for random shifts in allele frequencies and extinction. We provide the details of the algorithm in the Methods and the complete code is available on GitHub https://github.com/tecoevo/JohnsongrassDynamics for reproducibility.

## Results

### Genetic complexities

#### Standing genetic variation

We estimate the standing genetic variation for target-site resistance in untreated Johnsongrass by vector **v** in equation (22). The obtained population composition provided the initial seed bank and rhizomes composition for our deterministic population dynamics and corresponding stochastic simulations. We explore how the standing genetic variation for target-site resistance depends on the associated fitness cost (*c*) and its dominance (*k*_*c*_).

**Fig. 2:**
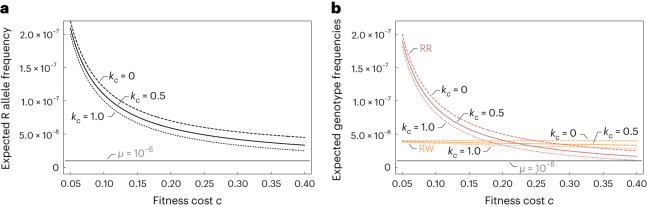
Variation of the approximated standing genetic variation for target-site resistance with the resistance cost. The genetic composition of untreated Johnsongrass populations is approximated on the basis of equation (22) and shown for different degrees of dominance (*k*_*c*_) of the resistance cost. Dashed lines correspond to a recessive resistance cost (*k*_*c*_ = 0), solid lines indicate partial dominance (*k*_*c*_ = 0.5) and dotted lines complete dominance (*k*_*c*_ = 1). The grey line marks the spontaneous mutation rate of resistance alleles (*μ* = 10^−8^). **a**, Expected frequency of the resistance allele R in an untreated population depending on the resistance cost (*c*). **b**, Expected frequencies of the resistant genotypes in an untreated population as a function of the resistance cost (*c*). The frequency of resistant heterozygotes (RW) is shown in yellow and resistant homozygotes (RR) are represented in red.

The frequency of resistance alleles R is controlled by the fitness cost on seed production (Fig. 2a). The frequency decreases with an increasing fitness cost. For fecundity reductions in resistant homozygotes exceeding 12%, we expect a resistance allele frequency in the order of the considered de novo mutation rate *μ* = 10^−8^ (refs. ^37,38^); indicating that competition with the sensitive type prevents the establishment of the resistant mutants. A higher degree of dominance regarding the fitness cost decreases the R allele fraction slightly, which is less visible for small fitness costs. Likewise, the frequency of both resistant types is slightly reduced by a higher dominance of the fitness cost (Fig. 2b). Remarkably, the expected frequency of heterozygotes shows little variation with the fitness cost. However, the expected frequency of resistant homozygotes increases with a decreasing fitness cost, most extreme for minimal fitness costs. This differential behaviour is because Johnsongrass is predominantly self-pollinated. Self-fertilized heterozygous plants (95%) produce only one-half of heterozygous seeds and one-quarter of each homozygote. Therefore, we see an increased level of homozygosity under low resistance costs. For costs lower than 20–25% (depending on the degree of dominance), we expect homozygous-resistant plants to be more abundant than heterozygotes.

In natural populations of annual ryegrass, plants with target-site resistance against an ALS-inhibitor were found at a frequency ranging from 1 × 10^−5^ to 1.2 × 10^−4^ (ref. ^39^). In another study one individual carrying a specific ACCase target-site resistance was detected in a sample of 685 blackgrass (*Alopecurus myosuroides*) specimens collected before the introduction of ACCase-inhibiting herbicides^28^. This abundance corresponds to a resistance allele frequency of 7.3 × 10^−4^. According to our model, we expect resistance alleles and resistant individuals in untreated fields at a frequency of 1 × 10^−5^ for a 0.1% fitness cost on seed production (data available on GitHub at https://github.com/tecoevo/JohnsongrassDynamics/tree/main/Fig2/data). Such a low fitness cost might not be detectable. Indeed, no associated fitness cost was detected for two out of three mutant ACCase alleles investigated in blackgrass^40^. However, for a fitness cost on seed production of 30%, reported for a mutant ACCase allele endowing herbicide resistance in Johnsongrass^30^, the expected resistance allele frequency and cumulative frequency of resistant types range from 3.3 × 10^−8^ to 5.3 ×10^−8^ and 4.9 × 10^−8^ to 7.3 × 10^−8^, respectively, depending on the dominance of this fitness cost. This initial prevalence of target-site resistance is in the order of the spontaneous mutation rate^37,38^ and considerably lower than what is reported in the literature^28,39^.

#### Resistance allele dominance and fitness cost dominance

An increase in the dominance of the resistance cost leads to a relatively small reduction in the overall fitness of heterozygotes, resulting in a minor decrease in the eventual abundance of heterozygotes and sensitive homozygotes (Supplementary Fig. 1). Sensitive plants decrease in abundance with heterozygous plants due to the prevalence of self-pollination. Reduced resistance dominance drastically lowers the fitness of heterozygotes under herbicide application. The reduced survival strongly decreases the number of heterozygous and homozygous sensitive plants in the population.

A high dominance of resistance accelerates target-site resistance evolution when resistance is rare while hindering fixation of the resistance allele (Supplementary Fig. 3a). At low frequencies of target-site resistance, it is advantageous if heterozygotes have a high fitness and produce many offspring, of which most are resistant again. This, in turn, is a disadvantage for allele fixation since the sensitive allele is masked in heterozygotes.

#### Fitness cost

Using stochastic simulations, we investigate the impact of resistance cost on target-site resistance evolution and resulting population regrowth in herbicide-treated Johnsongrass. It is worth noting that the fitness cost determines the frequency of standing genetic variants, substantially affecting the probability of Johnsongrass escaping from control and regrowing.

**Fig. 3:**
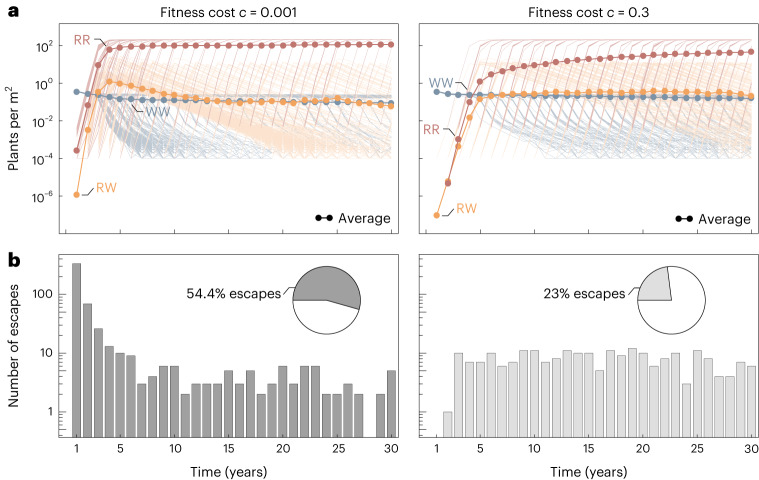
Simulated target-site resistance evolution and resulting population regrowth in herbicide-treated Johnsongrass for low and high resistance cost. Shown are the results of 1,000 simulation runs obtained for a partially dominant resistance allele (*k*_*h*_ = 0.5) and fitness cost (*k*_*c*_ = 0.5). The initial genotype composition differs between the low (*c* = 0.001) and high (*c* = 0.3) fitness cost (compare Fig. 2b). **a**, Changes in genotype composition of plants ($\widetilde{P}$) over 30 years of herbicide application for low and high resistance cost. The frequency of sensitive plants (WW) is shown in blue, resistant heterozygotes (RW) in yellow and resistant homozygotes (RR) in red. The thick lines with closed circles correspond to the average of all simulation runs and the thin lines represent the individual realizations. **b**, Distribution of escapes from control over 30 years of herbicide application for low and high resistance cost. Weed populations can regrow under herbicide treatment if a resistant plant establishes on the field and reproduces. The year of escape from control is the year in which the first homozygous-resistant plant survives until reproduction. The pie charts display the proportion of simulation runs where the weed population escapes from control and regrows.

Presuming that the resistant types manage to establish, the change in population composition is slightly faster under a low resistance cost on seed production (*c* = 0.001) compared to a high cost (*c* = 0.3) (Fig. 3a). The resistant homozygotes outcompete other types more easily (see also Supplementary Fig. 3b). Moreover, with a low fitness cost of 0.1%, the population escapes control by the herbicide in over half of the simulation runs (Fig. 3b). Most of these simulated populations start to grow again within the first years (78.5% within the first 3 years and 84.4% within 6 years), where the year of escape from control and the start of regrowth is defined as the year in which the first homozygous-resistant plants survive till reproduction. This resurgence is due to a high initial resistance allele frequency under low fitness costs (compare Fig. 2). Thus, resistant individuals will probably be in the field at the start of treatment. The impact of the fitness cost itself is comparably low. Under herbicide application, resistant plants have a major selective advantage over sensitive plants, even with a high fitness cost on seed production. For a higher fitness cost of 30%, less than a quarter of simulated populations escape from control by the herbicide and regrow within 30 years (Fig. 3b). The distribution of escapes from control is less skewed and more uniform, with almost no escapes in the first 2 years. Due to a low initial frequency of resistant types (compare Fig. 2), the population rescue depends on the probability of a new mutation arising or resistant seeds in the seed bank germinating.

#### Sexual reproduction

Genetic diversity mainly results from sexual reproduction, while perennials of a well-adapted type rapidly spread through asexual propagation. Two characteristics of the sexual reproduction of Johnsongrass, namely self-pollination and seed bank formation, seem particularly relevant for the population dynamics and target-site resistance evolution.

#### Self-pollination

**Fig. 4:**
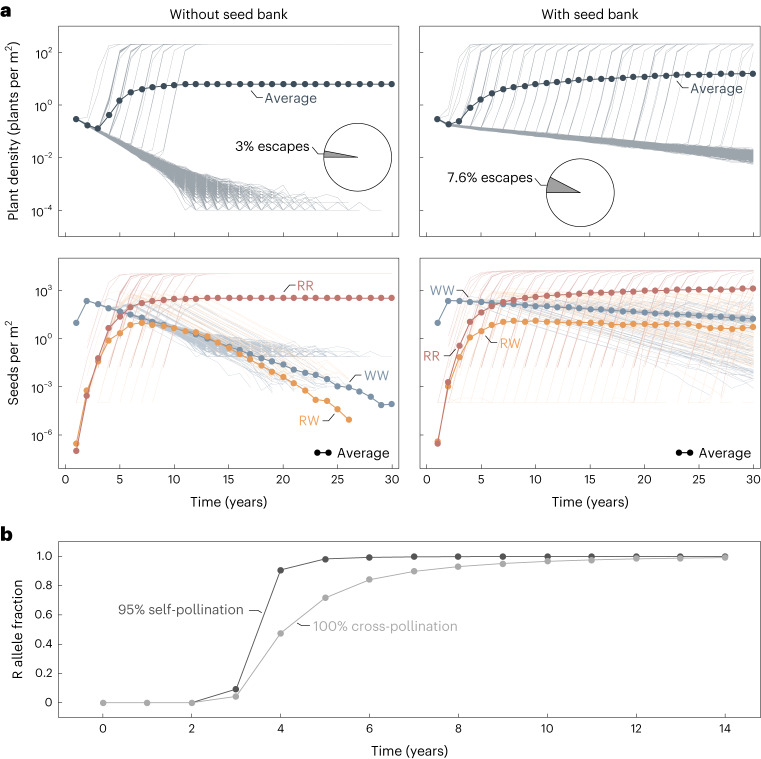
Predicted target-site resistance evolution in herbicide-treated Johnsongrass depending on seed bank formation and self-pollination. The results are obtained for a partially dominant resistance allele (*k*_*h*_ = 0.5) and fitness cost (*k*_*c*_ = 0.5). **a**, Simulated changes in Johnsongrass density ($\widetilde{P}/A$) and genotype composition of seeds (in the seed bank (*B*) if formed, otherwise produced seeds (*S*) that survived the winter) over 30 years of herbicide application and tillage depending on the formation of a seed bank. Shown are the results of 1,000 simulation runs. The thick lines with closed circles correspond to the average of all simulation runs and the thin lines represent the individual realizations. The frequency of sensitive seeds (WW) is shown in blue, resistant heterozygotes (RW) in yellow and resistant homozygotes (RR) in red. The pie charts display the proportion of simulation runs in which the weed population escapes from control and regrows due to herbicide resistance evolution. **b**, Predicted changes in the frequency of the resistance allele R in Johnsongrass plants ($\widetilde{P}$) under herbicide application for pure cross-pollination and 95% self-pollination. Shown are predictions of our deterministic model.

Selfing increases the level of homozygosity in the population. Hence, the heterozygotes are considerably less abundant compared to a solely cross-pollinated population (Supplementary Fig. 2) and the proportion of resistance alleles R increases faster under self-pollination (Fig. 4b). The interplay of a higher generation of homozygous-resistant individuals and the selective pressure exerted by the herbicide causes the accelerated target-site resistance evolution in self-pollinated weed populations. It might seem surprising that our model predicts an initial decline in homozygous-resistant seeds under herbicide application if the weed population is cross-pollinating. This decline is because, in our model, the initial population composition derives under the assumption of no cross-pollination. The sensitive type dominates over the first years, producing a high fraction of sensitive pollen. Therefore, in a solely cross-pollinated population, pollination with sensitive pollen prevails.

#### Seed bank

Most simulated populations that lack a seed bank go extinct within 30 years under herbicide application and tillage (Fig. 4a). At the same time, we see no extinctions within this period for simulated populations that include a seed bank. In the latter case, some resistant seeds might be left in the seed bank, even if the resistant types do not establish during the early years of herbicide application. The weed population thus has the potential to escape from control even after being controlled at very low densities for many years. Moreover, since populations with a seed bank are larger, the probability of mutations arising increases. Therefore, the distribution of escapes from control over time, observed in the stochastic simulations, is very wide for populations containing a seed bank. The escapes aggregate to 7.6% of the realizations. In contrast, in simulations without a seed bank, populations started regrowing solely in the first 9 years, accounting for 3% of the runs.

Without a seed bank, the sensitive type disappears from almost all simulated populations, while most seed banks still contain some sensitive seeds after 30 years of recurrent herbicide application and tillage. The seed bank composition changes more slowly than the genotype composition of plants on the field, preserving genetic variation (Supplementary Fig. 2). The seed bank only slightly delays the rise of resistance alleles in plants over several years of herbicide treatment (Supplementary Fig. 3c).

### Control strategies

#### Combined control regimes

**Fig. 5:**
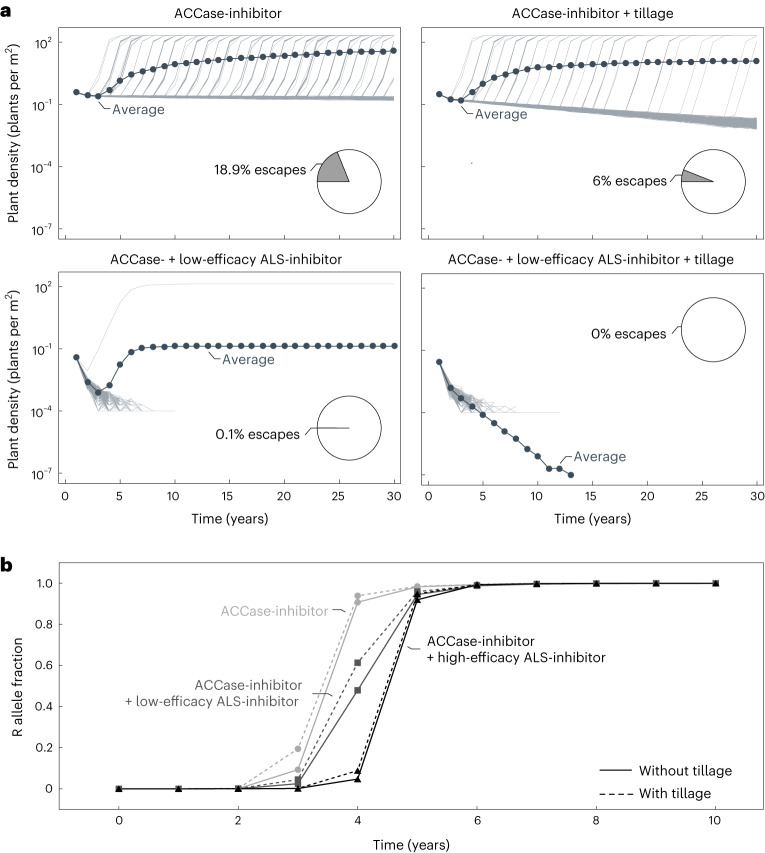
Predicted population dynamics and target-site resistance evolution in Johnsongrass under different control regimes. The results are obtained for a partially dominant resistance allele (*k*_*h*_ = 0.5) and fitness cost (*k*_*c*_ = 0.5). **a**, Simulated changes in Johnsongrass density ($\widetilde{P}/A$) and proportion of populations escaping from control over 30 years of different control regimes. Shown are the results of 1,000 simulation runs. The thick lines with closed circles correspond to the average of all simulation runs and the thin lines represent the individual realizations. The pie charts display the proportion of simulation runs in which the weed population escapes from control and regrows due to herbicide resistance evolution. The distinct control strategies are from left to right, top to bottom: ACCase-inhibitor application, ACCase-inhibitor application combined with tillage, application of ACCase-inhibitor and ALS-inhibitor with low efficacy, application of ACCase-inhibitor and ALS-inhibitor with low efficacy combined with tillage. **b**, Predicted changes in the frequency of the ACCase resistance allele R in Johnsongrass plants ($\widetilde{P}$) under different control regimes. Shown are predictions of our deterministic model. The distinct control strategies are: ACCase-inhibitor (light grey line with closed circles) or ACCase-inhibitor and ALS-inhibitor with low (dark grey line with closed squares) or high (black line with closed triangles) efficacy applied solely (solid line) or combined with tillage (dashed line).

We examined the impact of a binary herbicide mixture and the integration of soil tillage on population dynamics and target-site resistance evolution in Johnsongrass. The integration of tillage with herbicide application reduces the weed population size notably (Fig. 5a). This control improvement is caused by increased winter mortality of rhizomes and enhanced herbicide efficacy on rhizome shoots. Therefore, mutations are less likely to arise and the proportion of simulation runs where the weed population regrows from resistant individuals also reduces. Nevertheless, the increased selective pressure with tillage causes the frequency of resistance alleles in the population to increase slightly faster than under herbicide application alone (Fig. 5b). Our result contradicts the finding of an earlier theoretical study that tillage delays the evolution of target-site resistance^13^. However, we could qualitatively reproduce their result using a similar parameter set (Supplementary Fig. 4). Therefore, we acknowledge the deviation due to the considerably lower reproductive capacity implemented in the earlier study^13^. The limited propagation increases the effect of higher rhizome mortality, overcoming the increased selective pressure on shoots under low frequencies of the resistance allele.

Adding a second herbicide with a reduced efficacy makes the simulated populations go extinct within 15 years and escapes very unlikely (Fig. 5a). Furthermore, the second herbicide delays the resistance evolution against the first herbicide (Fig. 5b). The reason is the inherent ecology of Johnsongrasses. As the herbicide efficacy is higher on seedlings than on rhizome shoots, the proportion of surviving plants originating from rhizomes increases when a second herbicide with a different mode of action is added. The selective pressure on these rhizome plants is lower than on plants that emerge from seeds. This effect is even more substantial for higher efficacies of the second herbicide. However, suppose a herbicide with reduced efficacy is applied individually. In that case, the exceptionally high reproductive capacity of Johnsongrass still allows the population to grow (Supplementary Fig. 5). The high density, in turn, leads to de novo mutations conferring herbicide resistance.

#### Herbicide rotation

**Fig. 6:**
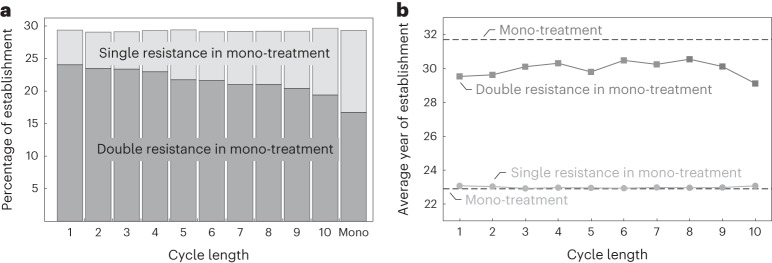
Simulated evolution of target-site resistance in Johnsongrass under binary herbicide rotations and mono-treatment. Shown are results of 10^5^ simulation runs obtained for a partially dominant resistance allele (*k*_*h*_ = 0.5) and fitness cost (*k*_*c*_ = 0.5). Considered are rotations of two herbicides with distinct target sites, ACCase- and ALS-inhibitors, that Johnsongrass is known to develop target-site resistance towards. We assume equal efficacy here. The cycle length refers to the number of years that one herbicide is recurrently applied before the treatment switches to the other herbicide. **a**, Proportion of simulated populations with resistant plants establishing on the field within 30 years of binary herbicide rotations depending on the cycle length and compared to mono-treatment. Bars show the percentage of simulation runs in which the plants ($\widetilde{P}$) evolve resistance, with the light grey part referring to the population with single resistance, that is target-site resistance against only one of the herbicides and the dark grey part reflecting double resistance. **b**, Average year of resistant plants establishing on the field within 30 years of binary herbicide rotations, depending on the cycle length and compared to mono-treatment. The light grey line with closed circles displays the average year the first resistant plant survives till reproduction and the dark grey line with closed squares depicts the average year of double-resistant plants establishing on the field. The dashed lines show the reflective year of resistance occurrence under mono-treatment.

We further investigated the effect of the cycle length in binary herbicide rotations on the establishment of resistant plants on the field (Fig. 6). We consider rotations of two herbicides with different target sites and equal efficacies, where mixture and mono-treatment can be viewed as the two extremes. Cycle length refers to the number of years one herbicide is applied recurrently before treatment switches to the other herbicide.

Herbicide rotations fail to reduce the probability of resistant plants establishing on the field. Varying slightly around 29.3% observed in mono-treatment, the proportion of simulated populations with resistant plants is not dependent on the cycle length (Fig. 6a). Due to the rapid population decrease, resistance evolves only in 0.02% of the plants treated with a mixture of the two herbicides. In contrast, the emergence of double resistance decreases with increasing cycle length from 24% in annual to 19.4% in decennial rotation. In mono-treatment, we observe double-resistant plants in 16.7% of the simulated populations and none under the application of herbicide mixture. Resistance against one of the herbicides in a binary rotation is sufficient for the weed population to grow in the long run (compare Supplementary Fig. 6). Further, herbicide rotations control sensitive plants with the same efficacy as a mono-treatment, leading to a similar probability of resistance adaptation. However, double resistance is costly for the plants, increasing the fitness cost on seed production in our model to 51% compared to 30% in single-resistant plants. When a herbicide is not applied, plants sensitive to this herbicide have a higher reproductive capacity. Therefore, less frequent switching of herbicides reduces the risk of double resistance.

In our simulations, plants with single resistance established before the potential occurrence of double resistance. On average, the first resistant plants establish on the field after about 23 years of herbicide application, unaffected by cycling (Fig. 6b). Under herbicide mixtures, double resistance occurs on average 6–7 years afterwards, about 2 years earlier than under mono-treatment. Nevertheless, rotations of the herbicides can delay the resistance allele fixation due to the alternating selective pressure and the fitness cost associated with resistance (compare Supplementary Fig. 7). Moreover, herbicide rotations delay weed population regrowth as compared to the application of a single herbicide (compare Fig. 5b and Supplementary Fig. 6). The average density reached by resistant populations after 50 years is 124–174 plants per m^2^ for herbicide rotations, considerably lower than 203 plants per m^2^ for mono-treatment.

## Discussion

Through developing a comprehensive life-cycle model of Johnsongrass, we have addressed the goals set out in our introduction. We showed (1) that standing genetic variation for target-site resistance is determined by the resistance cost, while its dominance has a minor impact; (2) how high resistance dominance hastens the initial spread of resistance alleles but delays fixation due to the masking of sensitive alleles in heterozygotes; and the resistance cost affects probability and time of control escapes mainly by determining the standing genetic variation, with little effect of its dominance; (3) that target-site resistance evolution is faster under self-pollination and a seed bank can increase the probability of escape from control while maintaining genetic variation; (4) that the integration of tillage with herbicide applications effectively reduces weed density and, thereby, the probability of escape from the control without delaying resistance evolution; herbicide mixtures not only control Johnsongrass more effectively but also delay target-site resistance evolution due to the weeds inherent ecology; and herbicide mixtures do not delay the onset of resistance but can slow resistance evolution and delay weed population regrowth. Beyond the specific implications for Johnsongrass control, our results have implications in the broader context of the essential concepts in controlling weeds: life-cycle details, chemical and physical control methods, resistance evolution and the prospects of ensuring food security. Below we draw out these conclusions.

Our analysis suggests that target-site resistance is associated with a meagre, probably undetectable, fitness cost when found in untreated populations at frequencies two orders of magnitude higher than the spontaneous mutation rate. For fitness costs exceeding 12% on seed production, the resistance allele frequency in populations that never encountered the herbicide before is expected to be similar in magnitude to the mutation rate. Not all mutations endowing target-site resistance in weeds are known to involve a fitness cost^40^. Furthermore, target-site resistance against ALS- and ACCase-inhibitors were found with a natural frequency in the order of 10^−5^ to 10^−4^ (refs. ^28,39^). According to our results, this would correspond to a meagre fitness cost. These findings coincide with the conclusions of ref. ^39^. It is, therefore, likely that in Johnsongrass, target-site mutations exist with very low or even no fitness cost. In this case, our simulations may underestimate the speed of resistance evolution and the risk of weed control failure.

We show a more than doubled probability of weed populations escaping herbicide control under a low fitness cost of 0.1% compared to a fitness cost of 30% on seed production. The latter cost was reported for a mutant ACCase allele endowing resistance in Johnsongrass^30^. The expected change in population composition is faster under low resistance costs and resistant plants establish earlier on the field, leading to population recovery. Thus, if the fitness cost of target-site resistance is low, control of the weed population with a single herbicide might fail quickly and should be avoided. Applying herbicide mixtures might be a good strategy in such a case and integrating other control measures such as tillage^5,8^.

Our results suggest that the fitness cost dominance has a minor impact on the prevalence of resistance and population dynamics under control. Unlike the fitness cost of resistance, herbicide resistance itself has no effect on the weed population in the absence of herbicides. The dominance of resistance, therefore, does not affect the standing genetic variation. However, it determines the fitness of heterozygotes under herbicide application, their abundance and the abundance of the sensitive type. A high dominance of resistance initially increases the speed of resistance evolution. However, later the effect reverses due to the masking of sensitive alleles in heterozygotes, delaying resistance evolution. This effect is observed for diploids and tetraploid perennials^14^.

Especially, target-site resistance alleles spread faster in haploids than in tetraploids since tetraploid plants need to acquire twice as many mutations to reach the same proportion of resistance allele^14^. However, in Johnsongrass, the W2027C target-site mutation was not found to be present on more than two ACCase alleles^6^. The cause might be a fitness penalty or the absence of homologous recombination between the two genomes of Johnsongrass^6^.

Our results indicate that target-site resistance evolution is faster in self-pollinating weed populations than in cross-pollinating populations. The increased homozygosity, combined with the selective pressure of the herbicide, causes a more rapid increase in the relative frequency of resistance alleles given resistant plants established. The higher number of homozygous-resistant individuals in the standing genetic variation of self-pollinated weeds comes at the cost of a lower number of resistance-carrying individuals, decreasing the probability of resistance adaptation for dominant target-site resistance. In another simulation study, populations of seed-propagated annual weeds needed to be larger for evolving resistance if they were self-pollinated compared to purely cross-pollinated populations^8^. However, the opposite is true for recessive target-site resistance. As heterozygotes are fully susceptible, recessive target-site resistance is more likely to establish in a herbicide-treated field if the weed is self-pollinated. We derived our initial composition of seeds and rhizomes from equation (22) under the assumption of pure self-pollination. This genotype composition was used to calculate the population dynamics in an outcrossing population. However, we obtained similar results with a control-free initialization phase of 30 years, allowing the population composition to stabilize under the assumed level of self-pollination.

Our simulations show that a seed bank notably delays the extinction of a weed population under control. A seed bank preserves genetic variation and considerably increases the establishment probability of resistant mutants, leading to population regrowth. Therefore, we emphasize the necessity of considering the details of sexual reproduction and seed bank during risk assessment of control measures. It has been argued that herbicide resistance spreads mainly through asexual propagation^13^. Once resistant plants have established, more resistant offspring might be generated via asexual propagation than seed production, considering the resistance cost on seed production and the possibility that produced seeds are not resistant. Resistance, however, generally originates in the seeds since mutations are most likely to arise during sexual reproduction. Even if standing genetic variants are present in the population, the number of seeds is considerably higher than that of rhizomes. Therefore, resistant individuals are more likely to be in the seed bank. Moreover, sexual reproduction is needed to generate homozygous-resistant offspring from heterozygous plants. We conclude that controlling the seed bank is essential in managing seed- and rhizome-propagated perennial weeds like Johnsongrass.

Integrating soil tillage with herbicide application further reduces Johnsongrass populations. We see no delay in the expected evolution of target-site resistance, as observed in an earlier study^13^. Nevertheless, the probability of Johnsongrass regrowth is more than halved by the reduction in population size. This result agrees with field studies of Johnsongrass^34^ and blackgrass^10^. However, tillage increases the risk of soil erosion^41^ and diminishes soil water^42^. Our study does not consider the risk of spreading resistant rhizomes and seeds with contaminated machinery.

Our results show that herbicide mixtures control Johnsongrass most effectively, with minimal risk of weed populations escaping control. Including a second herbicide delays resistance evolution, with an increased effect for higher herbicide efficacies. These results agree with other simulation studies, demonstrating that herbicide mixtures effectively control resistance evolution and are superior to rotations^8,9^. These conclusions were derived assuming independent action of the herbicides, no cross-resistance and full application rates. Applying two herbicides at full rates includes a higher economic cost than a mono-treatment and increases the environmental impact. Also, possible mechanisms of cross-resistance and synergistic or antagonistic interactions of the respective herbicides need to be explicitly considered. A comprehensive understanding of the effect of herbicide mixtures could be attained by incorporating herbicide interactions and cross-resistance into the present model and investigating different application rates and ratios.

In our simulations, binary herbicide rotations failed to reduce the risk of target-site resistance compared to mono-treatments. Frequent herbicide switching even favours the evolution of double resistance. Due to the tremendous reproductive capacity of Johnsongrass, subpopulations resistant to only one of the herbicides will, in our model, still grow over a rotation period with equal application of both herbicides. Although not delaying the onset of resistance, herbicide mixtures can slow resistance adaptation. This aspect, however, only takes effect if a relevant cost of resistance induces a selective disadvantage of the resistant types in seasons the respective herbicide is not applied. Our findings agree with a recent simulation study, which found that binary rotations only delay resistance evolution if a fitness cost is assumed^9^. However, they found more complex rotations, including four herbicides, to be more effective. Also, target-site resistance is not necessarily associated with a considerable fitness cost^40^. Moreover, rotations of two herbicides delay weed population regrowth. A field experiment on blackgrass found an average reduction in density of 59% after 7 years for yearly rotation of herbicide site of action compared to mono-treatment^10^. Our results show agreement with an earlier simulation study, in which rotations of herbicides were by far inferior to mixtures in controlling regrowth and herbicide resistance evolution^8^. Overall our results promise an advantage of herbicide rotations over mono-treatment in terms of slowing population regrowth and target-site resistance evolution, presuming a fitness penalty, with no consistent advantage of fast or slow rotation. However, rotations do not reduce the resistance risk. Fast rotations even favour the occurrence of double resistance. In general, combinations of herbicides are far more effective in reducing herbicide resistance evolution and control failure.

Crop rotations, especially when they incorporate highly competitive crops like maize, can further reduce the population size of Johnsongrass^10,43,44^. Other studies have focused on the effect of crop rotations along with rotations of herbicides in a theoretical attempt^14^. They highlight the effectiveness of a combination of crop rotation and rotation of herbicide classes in controlling Johnsongrass and delaying the evolution of herbicide resistance. However, they included resistance against only one of the herbicides, resulting from gene flow between resistant cultivated *Sorghum* and its wild relative. Our model can be extended to study the effect of crop rotations in terms of competition with the crop^45^. This extension can be achieved by adding the corresponding competition terms in the equations for density-dependant mortality (equation (9)) and reproduction (equations (11) and (12)).

Besides standing genetic variation and de novo mutations, resistance can also arise from pollen-mediated gene flow from neighbouring resistant populations or closely related resistant crops and the introduction of herbicide-resistant rhizomes and seeds via contaminated equipment^46,47^. One theoretical study included seed immigration and gene flow from pollen in their population model of annual weeds^48^, while a very recent study modelled resistance evolution in Johnsongrass with gene flow from resistant *Sorghum*^14^. Extending our model to a landscape scale with intra- and inter-specific gene flow will comprehensively assess resistance evolution in herbicide-treated perennial weeds.

Overall, we have presented a theoretical framework capable of capturing the complex life cycle of perennial plants. The framework can be modified to model specific cases, as we have shown for Johnsongrass and extended to answer pertinent questions from the points of view of different stakeholders in sustainable agriculture. For example, weed control measures always come with economic and socioecological facets. Thus, combining weed control and the associated socioeconomics in a single theoretical framework would be a fully functional tool built on the foundation of our current model.

## Methods

Rooted in the biology of Johnsongrass our model describes the dynamics of a weed population growing in one field. We capture the perennial life cycle by considering five life-history stages: seeds, seedlings, rhizomes, shoots and adult plants. One time step of the model corresponds to one year. We implement stochastic simulations of this population-based model capturing the stochastic nature of mutations, genetic drift and extinctions. In this section, we describe the equations reflecting the plant reproduction and management interventions, as well as the stochastic simulations. We provide the derivation of the model parameters in the Supplementary Information and Supplementary Table 1, featuring a parameter list and brief explanations.

### Life cycle

Johnsongrass plants are dormant throughout the winter, overwintering as seeds or rhizomes in the ground^4^. At the beginning of a growing season, seeds germinate to produce seedlings and shoots emerge from nodes on the rhizomes^5^. Mature plants produce new rhizomes and, after flowering, viable seeds^4,5^. Figure 1 illustrates the life cycle of Johnsongrass and its implementation in the model with both reproduction pathways, sexual via seeds and asexual through rhizomes.

Initially, we neglect herbicide resistance and its genetics and concentrate solely on the plant life cycle. Rhizomes in the ground at the beginning of season *t* are denoted by *Z*(*t*). Not all axillary buds formed on the nodes of a rhizome develop as shoots; some develop into secondary rhizomes or stay dormant^16,17^. Let *b* be the number of nodes on a rhizome and *g*_*Z*_ the proportion of nodes producing shoots during a season. Then the number of shoots (*T*(*t*)) at time *t* is given by,

1 $$T(t)={g}_{Z}\,b\,Z(t).$$

To begin with, we assume a constant seed bank *B* present in each season. Within a season *t*, only a certain proportion *g* of the seeds in the seed bank germinates to produce seedlings (*L*(*t*))^19^,

2 $$L(t)=g\,B.$$

Seedlings and shoots grow up to adult plants (*P*). We do not distinguish adult plants by their origin from seeds or rhizomes as they show similar growth characteristics^20,21^. Therefore,

3 $$P(t)=L(t)+T(t),$$

gives the number of adult plants (*P*(*t*)) present in season *t*. Plants form new rhizomes of which only a certain amount (1 − *d*_*Z*_) survives the winter to become the rhizomes of the next season *t* + 1,

4 $$Z(t+1)=(1-{d}_{Z})\,P(t).$$

### Control and resistance

Our model includes herbicide application and soil tillage as weed management techniques. We consider target-site resistance endowed by a single resistance allele R in a diploid genome. We include spontaneous mutations to the resistance allele R in sexual reproduction but no back mutations. We assume that the homozygous-resistant genotype confers complete resistance against the herbicide. The factor 0 ≤ *k*_*h*_ ≤ 1 captures the dominance of resistance such that the herbicide controls WW plants with efficacy *h* and RW plants with efficacy (1 − *k*_*h*_)*h*. Due to incomplete control of the rhizomes by herbicides and resprouting from dormant rhizome buds, the herbicide efficacy on rhizome shoots *h*_*T*_ is reduced compared to the herbicide efficacy on seedlings *h*_*L*_ (ref. ^32^). Soil tillage controls Johnsongrass rhizomes by exposing the fragments to low temperatures during winter, increasing mortality ${d}_{Z}^{* }$ (ref. ^4^) and enhances herbicide control of rhizome shoots ${h}_{T}^{* }$ (refs. ^31,33^).

Let $${{{\bf{T}}}}(t)=\left({T}_{{{{\rm{WW}}}}}(t),{T}_{{{{\rm{RW}}}}}(t),{T}_{{{{\rm{RR}}}}}(t)\right)$$, $${{{\bf{L}}}}(t)=\left({L}_{{{{\rm{WW}}}}}(t),{L}_{{{{\rm{RW}}}}}(t),{L}_{{{{\rm{RR}}}}}(t)\right),$$$${{{\bf{P}}}}(t)=\left({P}_{{{{\rm{WW}}}}}(t),{P}_{{{{\rm{RW}}}}}(t),{P}_{{{{\rm{RR}}}}}(t)\right)$$ and $${{{\bf{Z}}}}(t)=\left({Z}_{{{{\rm{WW}}}}}(t),{Z}_{{{{\rm{RW}}}}}(t),{Z}_{{{{\rm{RR}}}}}(t)\right)$$ be the vectors of shoot, seedling, plant and rhizome numbers in growing season *t*, respectively, and $${{{\bf{B}}}}=\left({B}_{{{{\rm{WW}}}}},{B}_{{{{\rm{RW}}}}},{B}_{{{{\rm{RR}}}}}\right)$$ the vector describing the composition of the seed bank. The population dynamics under herbicide application are then described by

5 $${{{\bf{T}}}}(t)={g}_{Z}\,b\,{{{\bf{Z}}}}(t),$$

6 $${{{\bf{L}}}}(t)=g\,{{{\bf{B}}}},$$

7 $${{{\bf{P}}}}(t)={{{\bf{L}}}}(t)\left(\begin{array}{ccc}1-{h}_{L}&0&0\\ 0&1-(1-{k}_{h})\,{h}_{L}&0\\ 0&0&1\end{array}\right)+{{{\bf{T}}}}(t)\left(\begin{array}{ccc}1-{h}_{T}&0&0\\ 0&1-(1-{k}_{h})\,{h}_{T}&0\\ 0&0&1\end{array}\right),$$

8 $${{{\bf{Z}}}}(t+1)=(1-{d}_{Z})\,{{{\bf{P}}}}(t).$$

The herbicide efficiencies *h*_*L*_ and *h*_*T*_ are zero in a herbicide-free scenario. Enhanced winter mortality of rhizomes ${d}_{Z}^{* }$ depicts the use of mechanical control. For the case of the combined treatment, the herbicide efficacy on rhizome shoots ${h}_{T}^{* }$ is additionally increased.

### Intraspecific competition

Intraspecific competition affects the survival and the formation of reproductive structures in Johnsongrass^3,23^. We describe the density-dependent mortality of Johnsongrass, resulting from intraspecific competition, by the hyperbolic self-thinning function

9 $$\widetilde{{{{\bf{P}}}}}(t)={{{\bf{P}}}}(t)\,{\left(1+m\frac{{n}_{P}(t)}{A}\right)}^{-1},$$

where $\widetilde{{{{\bf{P}}}}}(t)$ is the vector of plants surviving till the end of season *t*, *A* the field size, *n*_*P*_(*t*) = ∑_*i*∈{WW, RW, RR}_*P*_*i*_(*t*) the total number of plants in the field without self-thinning (might be interpreted as young plants about to experience competition) and *m*^−1^ gives the highest possible density of Johnsongrass after self-thinning^22^. Therefore,

10 $${{{\bf{Z}}}}(t+1)=(1-{d}_{Z})\,\widetilde{{{{\bf{P}}}}}(t),$$

gives now the vector of rhizomes present at the start of season *t* + 1.

Comprehensive competition experiments in Johnsongrass are missing. Therefore, we base the fertility functions on the assumption that the total seed and rhizome yield per m^2^ approaches a constant value at moderate to high densities^24^. Then the mean seed production (*f*(*t*)) and mean rhizome bud production (*b*(*t*)) per plant in season *t* can be described by

11 $$f(t)=f\,{\left(1+a\frac{{n}_{\widetilde{P}}(t)}{A}\right)}^{-1},$$

12 $$b(t)=b\,{\left(1+a\frac{{n}_{\widetilde{P}}(t)}{A}\right)}^{-1},$$

respectively, where *f* and *b* are the mean yields of an isolated plant, *a* is the area required by a plant to produce *f* seeds and *b* rhizome buds and ${n}_{\widetilde{P}}(t)={\sum }_{i\in \left\{{{\mathrm{WW}}, {\mathrm{RW}}, {\mathrm{RR}}}\right\}}{\widetilde{P}}_{i}(t)$ gives the total number of plants at the end of season *t* (after self-thinning)^49^. As the number of buds produced on a rhizome determines the number of shoots that can emerge from this rhizome in the next growing season,

13 $${{{\bf{T}}}}(t)={g}_{Z}\,b(t-1)\,{{{\bf{Z}}}}(t),$$

gives the shoots emerging in season *t*.

### Pollination and seed bank dynamics

We consider a fitness cost *c* on seed production associated with herbicide resistance^30^. A dominance factor 0 ≤ *k*_*c*_ ≤ 1 controls the dominance of this fitness cost, such that the fecundity of RR and RS plants is reduced by *c* and *k*_*c*_*c*, respectively, compared to the fecundity *f* of susceptible plants. Johnsongrass is primarily self-pollinated^4^. Let *p*_self_ denote the proportion of self-pollination. Then the number of seeds with genotype *i* (${S}_{i}^{\rm{self}}(t)$) produced during season *t* by self-pollinated plants can be calculated as,

14 $${S}_{i}^{\rm{self}}(t)={p}_{\rm{self}}\,f(t)\,\widetilde{{{{\bf{P}}}}}(t)\,{{{{\bf{m}}}}}^{i}\,\left(\begin{array}{ccc}1&0&0\\ 0&1-{k}_{c}\,c&0\\ 0&0&1-c\end{array}\right),$$

with

15 $${{{{\bf{m}}}}}^{i}={\left({M}_{{{{\rm{WW}}}}{{{\rm{WW}}}}}^{i},{M}_{{{{\rm{RW}}}}{{{\rm{RW}}}}}^{i},{M}_{{{{\rm{RR}}}}{{{\rm{RR}}}}}^{i}\right)}^{T},$$

where ${M}_{jk}^{i}$ gives the proportion of type *i* seeds produced by a plant of genotype *j* pollinated by type *k* pollen. The seed production follows Mendelian inheritance and includes spontaneous mutations to the resistance allele R occurring with probability *μ* (Supplementary Information). However, up to 5% of cross-pollination has been observed in fields with Johnsongrass plants growing at sufficiently small distances^4^. Over the *t*th season a number of

16 $${S}_{i}^{\rm{cross}}(t)=\frac{(1-{p}_{\rm{self}})\,f(t)}{{n}_{\widetilde{P}}(t)}\,\widetilde{{{{\bf{P}}}}}(t)\,{{{{M}}}}^{i}\,\widetilde{{{{\bf{P}}}}}{(t)}^{T}\,\left(\begin{array}{ccc}1&0&0\\ 0&1-{k}_{c}\,c&0\\ 0&0&1-c\end{array}\right)$$

type *i* seeds (${S}_{i}^{\rm{cross}}(t)$) are produced by cross-pollinated plants, where

17 $${{{{M}}}}^{i}=\left(\begin{array}{ccc}{M}_{{{{\rm{WW}}}}\,{{{\rm{WW}}}}}^{i}&{M}_{{{{\rm{WW}}}}\,{{{\rm{RW}}}}}^{i}&{M}_{{{{\rm{WW}}}}\,{{{\rm{RR}}}}}^{i}\\ {M}_{{{{\rm{RW}}}}\,{{{\rm{WW}}}}}^{i}&{M}_{{{{\rm{RW}}}}\,{{{\rm{RW}}}}}^{i}&{M}_{{{{\rm{RW}}}}\,{{{\rm{RR}}}}}^{i}\\ {M}_{{{{\rm{RR}}}}\,{{{\rm{WW}}}}}^{i}&{M}_{{{{\rm{RR}}}}\,{{{\rm{RW}}}}}^{i}&{M}_{{{{\rm{RR}}}}\,{{{\rm{RR}}}}}^{i}\\ \end{array}\right),$$

contains the proportions of type *i* seeds produced by the different matings, ${M}_{jk}^{i},j,k\in \{\,{{\mbox{WW, RW, RR}}}\}$. Adding up the seeds produced by self- and cross-pollinated plants gives the total number of genotype *i* seeds (*S*_*i*_(*t*)) produced in season *t*,

18 $$\begin{array}{rcl}{S}_{i}(t)&=&{S}_{i}^{\rm{self}}(t)+{S}_{i}^{\rm{cross}}(t)\\ &=&f(t)\,\widetilde{{{{\bf{P}}}}}(t)\,\left(\begin{array}{ccc}1&0&0\\ 0&1-{k}_{c}\,c&0\\ 0&0&1-c\end{array}\right)\,\left[\frac{1-{p}_{\rm{self}}}{{n}_{\widetilde{P}}(t)}\,{{{{M}}}}^{i}\,\widetilde{{{{\bf{P}}}}}{(t)}^{T}+{p}_{\rm{self}}\,{{{{\bf{m}}}}}^{i}\right].\end{array}$$

Johnsongrass seeds are dormant and form a seed bank in the ground^4,5^. Before new seeds enter the seed bank *B*, they are subject to postdispersal predation and might lose viability or decay till the beginning of the next season with probability *d*_*S*_ (ref. ^18^). Within a season *t*, only a certain proportion *g* of seeds in the seed bank (*B*(*t*)) germinates to produce seedlings (*L*(*t*)),

19 $${{{\bf{L}}}}(t)=g\,{{{\bf{B}}}}(t),$$

while non-germinated seeds may either be lost, due to decay and viability loss, with a probability *d*_*B*_, or survive to be part of next season’s seed bank^18^. Therefore, the seed bank of season *t* + 1 consists of non-germinated seeds from the previous seed bank and the seeds produced during season *t*,

20 $${{{\bf{B}}}}(t+1)=(1-{d}_{B})\,(1-g)\,{{{\bf{B}}}}(t)+(1-{d}_{S})\,{{{\bf{S}}}}(t).$$

### Standing genetic variation

The natural frequency of target-site resistance exceeds the rate at which these mutations spontaneously occur, suggesting that herbicide resistance adaptation primarily proceeds from standing genetic variants^27^. We derive a matrix model approximation of the entire life-cycle dynamics. We use this matrix model to calculate the expected standing genetic variation for target-site resistance depending on the fitness cost associated with resistance and its dominance.

Let ${{{\bf{P}}}}(t)=\left({P}_{{{{\rm{WW}}}}}(t),{P}_{{{{\rm{RW}}}}}(t),{P}_{{{{\rm{RR}}}}}(t)\right)$ and ${{{\bf{B}}}}(t)=\left({B}_{{{{\rm{WW}}}}}(t),{B}_{{{{\rm{RW}}}}}(t),{B}_{{{{\rm{RR}}}}}(t)\right)$ be the vectors of plant and seed numbers in season *t*, respectively, and let $\left({{{\bf{P}}}}(t),{{{\bf{B}}}}(t)\right)$ be the population vector containing plant and seed numbers of all genotypes. We assume an infinite density limit (no self-thinning) and maximum reproduction (*f*(*t*) = *f*, *b*(*t*) = *b*, ∀*t* ≥ 0). Further, we neglect cross-pollination (*p*_self_ = 1) here. Under these assumptions, the population dynamics of Johnsongrass can be approximated by

21 $$\begin{array}{rcl} &&\left( {{\mathbf{P}}}(t+1), {{\mathbf{B}}}(t+1)\right)\\ &&= \left( {{\mathbf{P}}}(t), {{\mathbf{B}}}(t)\right) \underbrace{\left(\begin{array}{rcl} g_Z \, (1-d_Z)\, b \, {{I}} + g \, (1-d_S) \, f \, {{D}} \,\widetilde{{{M}}} & (1-d_S) \, f \, {{D}} \,\widetilde{{{M}}}\\ g \, (1-d_{B}) \, (1-g) \, {{I}} & (1-d_{B}) \, (1-g) \, {{I}} \end{array}\right)}_{{=: {{E}}}}, \end{array}$$

where *I* is the identity matrix of size 3,

$${{{D}}}=\left(\begin{array}{ccc}1&0&0\\ 0&1-{k}_{c}\,c&0\\ 0&0&1-c\end{array}\right),$$

is the diagonal matrix incorporating the resistance cost on seed production and

$$\widetilde{{{{M}}}}=\left(\begin{array}{ccc}{(1-\mu )}^{2}&2\,\mu \,(1-\mu )&{\mu }^{2}\\ \frac{1}{4}\,{(1-\mu )}^{2}&\frac{1}{2}\,(1-{\mu }^{2})&\frac{1}{4}\,{(1+\mu )}^{2}\\ 0&0&1\end{array}\right),$$

is the inheritance matrix, assuming simple Mendelian inheritance.

We use the Perron–Frobenius Theorem (Supplementary Information) to derive the long-term population dynamics

22 $$\mathop{\lim }\limits_{t\to \infty }\frac{\left({{{\bf{P}}}}(t+1),{{{\bf{B}}}}(t+1)\right)}{{\rho }^{t}}=\left({{{\bf{P}}}}(1),{{{\bf{B}}}}(1)\right)\,{{{{\bf{u}}}}}^{T}{{{\bf{v}}}},$$

where *ρ* is the positive and simple eigenvalue of *E*, that is in absolute value the largest eigenvalue and **u** and **v** are the corresponding positive right and left eigenvectors^50^. Thus, in the long run, the population composition is approximately a real multiple of **v**, regardless of the initial population. Therefore, **v** gives us a rough estimate of the expected standing genetic variation for target-site resistance in a Johnsongrass population never exposed to the herbicide (Fig. 2).

### Stochastic simulations

As in the underlying deterministic model, we model the population dynamics in discrete time steps of one growing season. The numbers of rhizome buds and seeds produced in season *t* by plants of a specific genotype *i* are drawn from Poisson distributions with mean *P*_*i*_(*t*)*b*(*t*) and *P*_*i*_(*t*)*f*(*t*), respectively. Probabilities, such as germination probability, death probabilities and herbicide efficacy, are realized on the population level for the different genotypes using binomial distributed random numbers. The number of sensitive seeds germinating in season *t* is, for example, given by a binomial random number with parameters *B*_WW_(*t*) and *g*. We use multinomial random numbers to derive the initial seeds and rhizomes and to model fertilization and mutation. Seeds resulting from self-pollination in heterozygous plants, for instance, are obtained from the multinomial distribution with the total number of seeds produced via self-pollination by heterozygous plants and the inheritance vector $\left(\frac{1}{4},\frac{1}{2},\frac{1}{4}\right)$ as parameters. For further details, we refer to the code available on GitHub.

### Reporting summary

Further information on research design is available in the Nature Portfolio Reporting Summary linked to this article.

## Supplementary information

Supplementary Information Supplementary text (model parameter derivation), Table 1 and Figs. 1–7.

Reporting Summary

## Data Availability

All data and plots generated in Mathematica v.13.2.0.0 are available on GitHub at https://github.com/tecoevo/JohnsongrassDynamics.

## Code availability

All code was written in MATLAB_R2021b and is available on GitHub at https://github.com/tecoevo/JohnsongrassDynamics.
