# Supplementary material for: Theoretical assessment of persistence and adaptation in weeds with complex life cycles
Source: Nat Plants. 2023 Aug 3;9(8):1267–79. doi: 10.1038/s41477-023-01482-1 (PMC10435386; doi:10.1038/s41477-023-01482-1)
Supplement: Supplementary file 1 — Supplementary text (model parameter derivation), Table 1 and Figs. 1–7. [file 41477_2023_1482_MOESM1_ESM.pdf]

nature plants

Article https://doi.org/10.1038/s41477-023-01482-1

Theoretical assessment of persistence and adaptation in weeds with complex life cycles

In the format provided by the authors and unedited

Supplementary Information: Theoretical assessment of persistence and adaptation in weeds with complex life cycles

Dana Lauenroth[1,*] & Chaitanya S. Gokhale[1,2]

[1]Research Group for Theoretical Models of Eco-evolutionary Dynamics,
Department Theoretical Biology, Max Planck Institute for Evolutionary Biology,
August-Thienemann-Straße 2, 24306 Plön, Germany

[2] Center for Computational and Theoretical Biology, Julius-Maximilians-Universität Würzburg,
Klara Oppenheimer Weg 32, 97070 Würzburg, Germany

[*] Corresponding Author; e-mail: lauenroth@evolbio.mpg.de

Contents

1 Model parameters **2**

1.1 Seed germination. 2
1.2 Rhizome bud sprouting. 2
1.3 Maximum plant density. 3
1.4 Seed survival. 3
1.5 Rhizome survival. 4
1.6 Herbicide efficacy. 5
1.7 Inheritance. 7

2 Matrix theory **7**

3 Supplementary figures **10**

1 Model parameters

1.1 Seed germination.

Germination of Johnsongrass seeds is significantly influenced by temperature, light and burial depth (Horowitz 1972, Tóth & Lehoczky 2006, Krenchinski et al. 2015). Keeley & Thullen (1979) observed germination frequencies in the range of 24 to 91.5 % within three weeks for monthly sowing from March to October. In another study, no seed germination was documented at temperatures under 19°C and a maximum of 64 % germination within 15 days was found at 39°C (Horowitz 1972). However, Krenchinski et al. (2015) reported considerably lower germination frequencies, lower optimal germination temperature and reduced germination in the absence of light. They documented maximum germination at 30°C of 18.50 and 13.75 % in the presence and absence of light, respectively. Seed germination of Johnsongrass decreases with increasing burial depth from reported 64 % germination from 1 cm depth to 6 % from a sowing depth of 25 cm (Tóth & Lehoczky 2006).

However, the population-level model does not account for these environmental factors. Warwick & Black (1983) found a mean seed germination frequency of 38 % in overwintering populations of Johnsongrass. This observation agrees with a study by Legutzamón (1986). Therein, 37 % of seeds left on the soil surface germinated within the first year and 25 % of seeds transferred from the surface to 15 cm depth in autumn, simulating tillage practice. Our germination probability $g = 0.3$ is between these values to account for seed burial due to natural causes or tillage. It is also consistent with the mean germination frequency documented by Egley & Chandler (1978).

1.2 Rhizome bud sprouting.

In Johnsongrass, the development of rhizome buds is markedly controlled by apical dominance (Beasley 1970, Hull 1970). Rhizome apexes and emerged shoots partially suppress growth from adjacent buds (Beasley 1970, Hull 1970). Moreover, on branched rhizomes, secondary rhizomes influence the sprouting of axillary buds on the primary rhizome in an inverse manner (Beasley 1970). Bud sprouting and shoot growth in Johnsongrass are reported to be highly temperature dependant (Hull 1970, Horowitz 1972), with a threshold temperature of 10°C, and to vary between populations (Loddo et al. 2012). In a study of Loddo et al. (2012) with 4-node rhizome segments, shoot emergence was highest at 16°C, with a between population average of 48 %. Over the temperature range of $10 - 24$°C, a total of 26 and 39 % of the rhizome nodes developed sprouts in the two studied populations. Hull (1970) documented an average bud germination on one-node rhizome pieces of 14 and 92 % at 15 and 30°C, respectively.

However, Keeley & Thullen (1979) detected no relevant influence of temperature on rhizome bud sprouting (56 % at $18 - 35$°C) in their experiments, but a significant impact on shoot development. Shoots emerged from 13 and 88 % of the sprouted buds at 18 and $24 - 35$°C, respectively.

Our sprouting probability for buds on fragmented rhizomes in a tillage regime is 30 %, in agreement with the data of Loddo et al. (2012). Apical meristems, emerged shoots and lateral rhizomes can inhibit axillary bud development on branched rhizomes (Hull 1970). Therefore, we assume a reduced sprouting probability of 20 % for no-tillage regimes.

1.3 Maximum plant density.

Johnsongrass produces tufts as a result of tillering, i.e. the development of secondary shoots from the crown area of existing plants (McWhorter 1971, Horowitz 1972). We do not explicitly model the production of tillers. However, the process of tillering is reflected in density-dependent reproduction. Lolas & Coble (1980) reported a considerable variation between different growing seasons in the number of tillers produced by a plant, ranging between 5 and 30 tillers per plant 60 days after planting, with an average of 19 tillers per plant grown from long rhizome pieces. Keeley & Thullen (1979) on average counted 5 to 20 tillers per plant, depending on the planting date. We assume that a plant not affected by intraspecific competition can, on average, produce 20 tillers.

In their experiments McWhorter (1971) observed an extreme variation between different Johnsongrass ecotypes in the number of culms growing in an area of one square meter, ranging from 65 up to 226 culms m^{-2}. In another study, 200 Johnsongrass culms were, on average, counted per m^2 in an untreated soybean field (Landry et al. 2016). Therefore we assume a possible highest density of 220 plants per m^2 after self-thinning from very high densities. However, the ceiling density reached in populations growing from low densities is lower.

Tillers contribute to the total culm density on the field. However, high densities delay or even inhibit tillering in Johnsongrass (Williams & Ingber 1977). We assume that intraspecific competition becomes appreciable at a density of 10 plants per m^2, i.e. 200 culms m^{-2} under maximum tillering. In the study of Keeley & Thullen (1979) Johnsongrass was planted at this density, meaning each plant had an area of 0.1 m^2 to produce the reported yields.

1.4 Seed survival.

Egley & Chandler (1978) documented no significant impact of the burial depth on seed longevity and an average of 62 % of seeds that were still viable after burial for 2.5 years. However, seeds were placed in polypropylene screen envelopes which prevented animal predation. It

was not recorded if unrecovered seeds decayed or germinated. In another study, likewise excluding predation and not tracking germination, more prolonged seed survival in deep layers (15 and 22.5 cm) as compared to seeds in shallow layers (soil surface and 7.5 cm) was noticed (Legutzamón 1986). After three years, they recovered less than $15~\%$ of viable seeds from the shallow layers, while 34.5 to $53.8~\%$ of seeds in the deeper layers were still viable. Nevertheless, regardless of the burial depth, virtually no viable seeds were found in their experiments after six years. Moreover, Legutzamón (1986) carried out one experiment in bare soil with different treatments. Some treatments comprised regular changes between the soil surface and 15 cm depth to examine the effect of cultivation on seed longevity. They found maximum seed survival to be less than 2.5 years. However, they discussed that the absence of vegetation in that experiment might have contributed to a faster loss of viable seeds. Bagavathiannan & Norsworthy (2013) reported 64 to $75~\%$ of seed loss due to predation, from dispersal in autumn till next spring, when seeds were left on the soil surface (no-tillage). Most recovered seeds $(78-91~\%)$ were buried in the soil. In the same period, they documented 6 to $17~\%$ seed decay. Also, a loss in viability of $23~\%$ was observed that increased to $52~\%$ (including germination) till the subsequent autumn. Overall the mean proportion of active seed bank still present in spring, after seed loss due to predation, decay and loss in viability, was 10 to $11~\%$ and reduced to 7 to $8~\%$ in autumn, after subsequent seed loss through decay, loss in viability and germination (Bagavathiannan & Norsworthy 2013). They documented no significant impact of residue cover or burial depth on seed decay and viability loss.

We consider only post-dispersal seed predation, as most surviving seeds are then buried in the soil and therefore subsequently inaccessible for predators, at a proportion of $75~\%$ (Bagavathiannan & Norsworthy 2013). Our model includes $17~\%$ seed decay and $23~\%$ loss in viability from the time of seed dispersal in autumn till next spring. We choose $24~\%$ and $31~\%$ yearly seed loss due to decay and loss in viability, respectively. With that, seed loss in our model is in the range reported by Bagavathiannan & Norsworthy (2013), but higher than found in the undisturbed experiments of Egley & Chandler (1978) and Legutzamón (1986). However, our parameters are in good agreement with the simulated cultivation experiments conducted by Legutzamón (1986). Bagavathiannan & Norsworthy (2013) calculated the proportion of predation, correcting for the proportion of decayed seeds. Following this, viable freshly produced seeds are lost to $0.94~\%$ $(1-(1-0.75-0.17)(1-0.23)\approx 0.94)$ in our model, and the yearly loss of viable seeds in the seed bank is $48~\%$ $(1-(1-0.24)(1-0.31)\approx 0.48)$.

1.5 Rhizome survival.

Winter mortality of Johnsongrass rhizomes is highly dependent on the soil temperature, determined by climatic conditions and burial depth (Stoller 1977, Warwick et al. 1986, Hartzler

et al. 1991). Stoller (1977) documented about 5 % of rhizomes surviving at a burial depth of 20 cm and no survival above 20 cm depth in the first winter with a temperature record of -9°C at 20 cm. In the second milder winter, more than 60 % of the rhizomes survived at 20 cm and rhizome survival increased with increasing depth in soil up to over 90 %. For overwintering populations, Warwick et al. (1986) reported a dramatic reduction of rhizome survival at 20 cm depth to 20 % in a more northern climate compared to 97 % survival under milder climatic conditions. They also recorded a survival dependency on burial depth, with only one out of 16 rhizomes surviving at a burial depth of 10 cm and 87.5 % winter survival at 30 and 50 cm. Hartzler et al. (1991) also found rhizome survival directly related to burial depth. With 5 and 25 % survival of 7.5 and 15 cm long rhizome pieces buried in soil 6 cm deep, survival of longer rhizome pieces was higher. Simultaneously, they did not notice a significant difference at 25 cm depth, where survival ranged between 85 and 87 %. Most rhizome biomass and nodes are found in the upper 30 cm, with the highest amount between 7.5 and 15 cm (Hartzler et al. 1991).

Our model does not account for the depth of rhizome burial or climatic conditions. We use the rhizome survival and distribution data published by Hartzler et al. (1991) to calculate the average winter mortality of long rhizome segments $d_Z = 0.35$, used in our model as the winter mortality in no-tillage regimes. Tillage cuts Johnsongrass rhizomes into smaller pieces and partly transports them to the soil surface. We assume that under control regimes comprising tillage, the share of rhizome pieces in the shallow layer is increased by 10 %. Together with the rhizome fragmentation, this leads to higher average winter mortality of $d_Z = 0.6$.

All non-germinated rhizomes rotted in the experiments of Warwick et al. (1986). Furthermore, after sprouting, the rhizomes of Johnsongrass disintegrate within 19 to 24 days (McWhorter 1961). Therefore we only consider winter survival and assume that rhizomes can only give rise to shoots in the subsequent growing season.

1.6 Herbicide efficacy.

The herbicide efficacy on Johnsongrass aboveground material depends on the developmental stage of the plants, with small young plants killed with higher efficacy than larger older plants (Tuesca et al. 1999, Dogan & Boz 2005, Johnson & Norsworthy 2014). The efficacy also varies between years due to environmental factors and application timing (Vidrine 1989, Tuesca et al. 1999).

Johnson et al. (2003) and Johnson & Norsworthy (2014) report the investigated ACCase-inhibitors to have a higher efficacy on Johnsongrass than ALS-inhibitors included in their studies. A single application of the ACCase-inhibitor Quizalofop led to a 97 % reduction in Johnsgrass biomass three weeks after treatment. In contrast, single applications of the

ALS-inhibitors Nicosulfuron and Primisulfuron reduced fresh weight biomass by only 88 % (Johnson et al. 2003). In the second study, a higher rate of the ACCase-inhibitor Clethodim controlled small plants (15 and 30 cm) at 97 % two weeks after treatment while Nicosulfuron provided 90 % control of 15 cm Johnsongrass and only 74 % control of 30 cm plants (Johnson & Norsworthy 2014). Even though they noticed Johnsongrass regrowth after two weeks, stand reduction four weeks after treatment, compared to an untreated control, was similar to the initial weed control.

Due to insufficient translocation of active ingredients and the inactive state of rhizome nodes, rhizomes are controlled inadequately (Beasley 1970). Regrowth from the latter may occur, resulting in a reduced herbicide efficacy on rhizome shoots (Beasley 1970, Tuesca et al. 1999). In a study of Vidrine (1989), the average control of Johnsongrass seedlings provided by different ACCase-inhibiting herbicides was 99 % three weeks after treatment and 95 % for rhizome shoots. Sethoxidim failed to control Johnsongrass shoots effectively in one year of the experiment. Excluding sethoxydim, the other three ACCase-inhibitors controlled seedlings and shoots at 99.8 and 98.5 %, respectively. Johnsongrass control was reduced 12 weeks after treatment. Nevertheless, with one exception, control was still at least 86 % and, in most cases, even above 90 %. Winton-Daniels et al. (1990) reported that regrowth occurred after all treatments with ACCase-inhibitors but that sequential treatments typically control Johnsongrass better than single applications. A second herbicide application later in the season reduces the time for Johnsongrass plants to regrow. Therefore, regrown plants might be unable to produce new rhizomes and viable seeds until the end of the growing season.

Given the above points, it is reasonable to assume that the final control provided by a sequential treatment is similar to the initial control a single application gives. As per Vidrine (1989), we choose the efficacy of ACCase-inhibiting herbicides to be 99.8 % on seedlings and to be reduced on shoots with 98.5 %. In some parts of our manuscript, this efficacy is also assumed for the ALS-inhibitor to secure effective weed reduction, for example, in herbicide rotations, and is denoted by 'high efficacy'. As ALS-inhibiting herbicides are reported to control Johnsongrass with a lower efficacy (Johnson et al. 2003, Johnson & Norsworthy 2014), we also investigate a low ALS-inhibitor efficacy of 92 and 90 % on seedlings and rhizome shoots, respectively, denoted by 'low efficacy'. Mechanical practices inducing increased bud activity as a response to rhizome fragmentation allow for improved control of rhizomes and shoots by herbicide application (Hull 1970, Tuesca et al. 1999). Therefore herbicide efficacy on shoots of fragmented rhizomes is assumed to be intermediate between seedlings and shoots on intact rhizomes.

1.7 Inheritance.

We suppose simple Mendelian inheritance. Mutations to the resistance allele R are assumed to arise with probability $\mu = 10^{-8}$, but back mutations are not considered (Haughn & Somerville 1987, Harms & DiMaio 1991). Therefore the inheritance matrices (Eq. 17) used to calculate the number of seeds arising from cross-pollination (Eq. (16)) are given by,

$$\mathbf{M}^{\mathrm{WW}} = (1-\mu)^2 \begin{pmatrix} 1 & 0.5 & 0 \\ 0.5 & 0.25 & 0 \\ 0 & 0 & 0 \end{pmatrix}, \tag{S.1}$$

$$\mathbf{M}^{\mathrm{RW}} = 2\,\mu\,(1-\mu) \begin{pmatrix} 1 & 0.5 & 0 \\ 0.5 & 0.25 & 0 \\ 0 & 0 & 0 \end{pmatrix} + (1-\mu) \begin{pmatrix} 0 & 0.5 & 1 \\ 0.5 & 0.5 & 0.5 \\ 1 & 0.5 & 0 \end{pmatrix}, \tag{S.2}$$

$$\mathbf{M}^{\mathrm{RR}} = \mu^2 \begin{pmatrix} 1 & 0.5 & 0 \\ 0.5 & 0.25 & 0 \\ 0 & 0 & 0 \end{pmatrix} + \mu \begin{pmatrix} 0 & 0.5 & 1 \\ 0.5 & 0.5 & 0.5 \\ 1 & 0.5 & 0 \end{pmatrix} + \begin{pmatrix} 0 & 0 & 0 \\ 0 & 0.25 & 0.5 \\ 0 & 0.5 & 1 \end{pmatrix}. \tag{S.3}$$

2 Matrix theory

We make use of a well-known result from the theory of matrices.

Theorem 1 (Perron-Frobenius (Harris 1963)) *Let* $\mathbf{A}$ *be a nonnegative* k *x* k *matrix, such that* $\mathbf{A}^N$ *is positive for some* $N \in \mathbb{N}$*. Then* $\mathbf{A}$ *has a positive and simple eigenvalue* ρ *that is in absolute value greater than any other eigenvalue of* $\mathbf{A}$*.* ρ *corresponds to positive right and left eigenvectors* $\mathbf{u}$ *and* $\mathbf{v}$*, which are the only nonnegative eigenvectors of* $\mathbf{A}$*. Moreover, for* $n \in \mathbb{N}$ *we have*

$$\mathbf{A}^n = \rho^n\,\mathbf{u}^T\mathbf{v} + \mathbf{R}^n\,, \tag{S.4}$$

with the normalisation $\mathbf{u}\,\mathbf{v}^T = 1$*. Furthermore,* $|\mathbf{R}^n| := \sum_{i,j=1}^{k} (R^n)_{ij} = \mathcal{O}(\alpha^n)$ *for some* α*,* $0 < \alpha < \rho$*.*

From Eq. (S.4) it follows that in the limit

$$\lim_{n\to\infty} \frac{\mathbf{A}^n}{\rho^n} = \mathbf{u}^T\mathbf{v}\,, \tag{S.5}$$

holds, i.e. $\mathbf{A}^n$ is dominated by ρ^n for $n \to \infty$.

Table S1: Model parameters, values and references.

**Model parameters**	**Value**	**Comments**	**References**
Sprouting probability<br>without tillage<br>after tillage	 $g_Z = 0.2$<br>$g_Z^* = 0.3$	influenced by apical dominance and temperature; wide variation in reported values (Hull 1970, Horowitz 1972, Keeley & Thullen 1979)	Loddo et al. (2012)
Germination probability	$g = 0.3$	influenced by environmental factors not considered in the model, e.g. temperature, light and burial depth (Horowitz 1972, Tóth & Lehoczky 2006, Krenchinski et al. 2015)	Egley & Chandler (1978), Warwick & Black (1983), Legutzamón (1986)
Fitness cost on seed production	$c = 0.3$	in blackgrass, only one mutation conferring resistance to ACCase-inhibitors was found to be associated with a fitness cost (Menchari et al. 2008)	Panozzo & Sattin (2021)
Proportion of self-pollination	$p_{self} = 0.95$	there are arguments for higher levels of cross-pollination (Ohadi et al. 2018)	Warwick & Black (1983)
Mutation rate	$\mu = 10^{-8}$	estimates of spontaneous mutation rates of ALS-resistance alleles in thale cress (*Arabidopsis thaliana*) and cultivated tobacco (*Nicotiana tabacum*)	Haughn & Somerville (1987), Harms & DiMaio (1991)
Possible highest limit of plant density (m^{-2})	$m^{-1} = 220$	a lower ceiling density is reached for populations growing from low densities	McWhorter (1971), Williams & Ingber (1977), Landry et al. (2016)
Herbicide efficacy<br>ACCase-inhibitor (high efficacy ALS-inhibitor)<br>on seedlings<br>on shoots<br>on shoots after tillage<br>ALS-inhibitor (low efficacy)<br>on seedlings<br>on shoots<br>on shoots after tillage	 <br>$h_L = 0.998$<br>$h_T = 0.985$<br>$h_T^* = 0.992$<br><br>$h_L = 0.92$<br>$h_T = 0.90$<br>$h_T^* = 0.91$	influenced by environmental factors and application timing (Tuesca et al. 1999, Dogan & Boz 2005, Johnson & Norsworthy 2014); wide variation in reported regrowth (Vidrine 1989, Johnson & Frans 1991)	Vidrine (1989)<br>Johnson et al. (2003)
Number of rhizome buds produced per isolated plant	$b = 140$	wide variation in reported numbers (Anderson et al. 1960, Hartzler et al. 1991, Acciaresi & Chidichimo 2005)	Keeley & Thullen (1979)

(continues)

Table S1: (continued)

Model parameters	Value	Comments	References
Rhizome loss<br>winter mortality<br>without tillage<br>after tillage	 <br>$d_Z = 0.35$<br>$d_Z^* = 0.6$	influenced by climatic conditions and burial depth not considered in the model (Stoller 1977, Warwick et al. 1986, Hartzler et al. 1991)	Hartzler et al. (1991)
Number of viable seeds produced per isolated plant	$f = 13,000$	wide variation between ecotypes and in reported numbers (McWhorter 1971, Ghersa et al. 1985, Acciaresi & Chidichimo 2005)	Keeley & Thullen (1979)
Seed loss<br>fresh seeds<br>old seeds in seed bank	 $d_S = 0.94$<br>$d_B = 0.48$	conflicting information on the impact of burial depth and wide variation in reported values (Egley & Chandler 1978, Legutzamón 1986, Bagavathiannan & Norsworthy	Bagavathiannan & Norsworthy (2013)
area (m^2) required by a plant to produce f seeds and b rhizome buds	$a = 0.1$	assuming intraspecific competition becomes appreciable at a density of 10 plants m^{-2}	Keeley & Thullen (1979)
Initial density (m^{-2})<br>seeds<br>rhizomes	 10<br>1		Liu et al. (2019)
Field size (m^2)	$A = 10,000$		Liu et al. (2019)

3 Supplementary figures

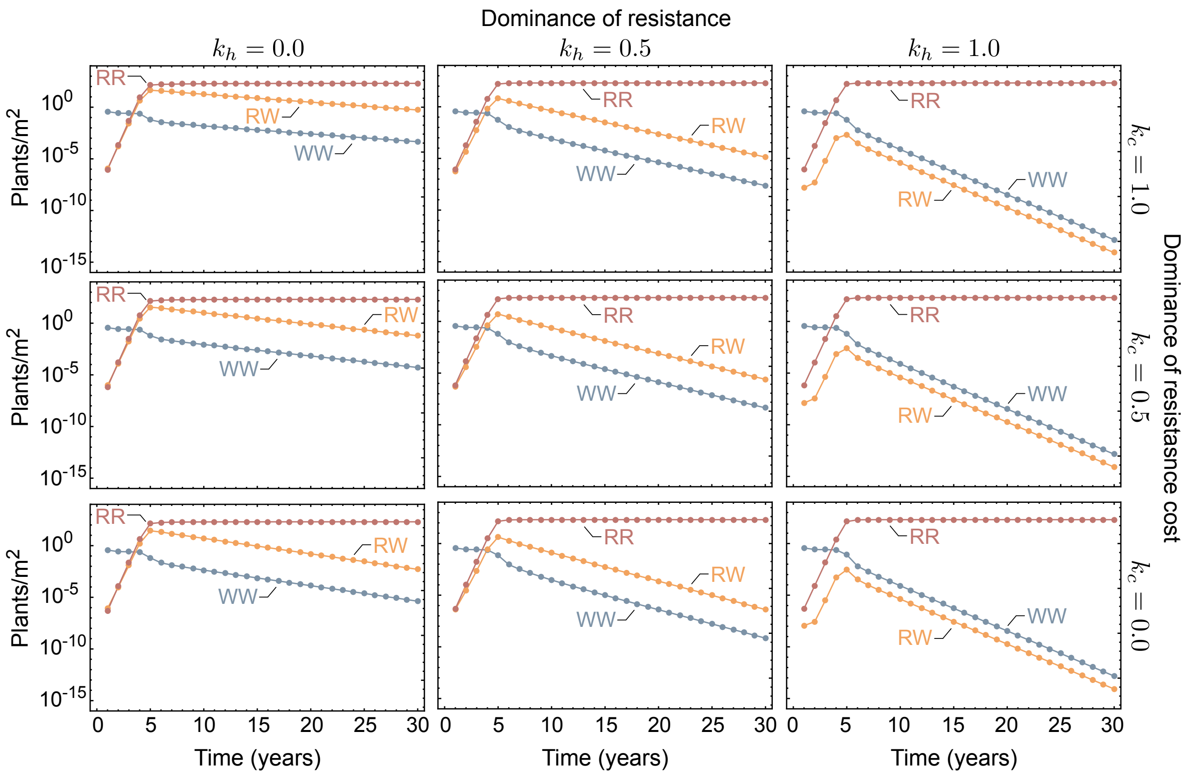

Fig. S1: **Predicted target-site resistance evolution in herbicide-treated Johnsongrass depending on the resistance and cost dominance.** Shown are predictions of our deterministic model for the genotype composition of Johnsongrass plants ($\widetilde{P}$) over 30 years of herbicide application for different degrees of dominance regarding resistance ($\mathbf{k_h}$) and the fitness cost associated with resistance ($\mathbf{k_c}$). ($k = 0$) corresponds to recessivity, ($k = 0.5$) indicates partial dominance and ($k = 1$) complete dominance. The frequency of sensitive plants (WW) is shown in blue, resistant heterozygotes (RW) in yellow and resistant homozygotes (RR) are represented in red. The initial genotype composition differs with the dominance of resistance and fitness cost.

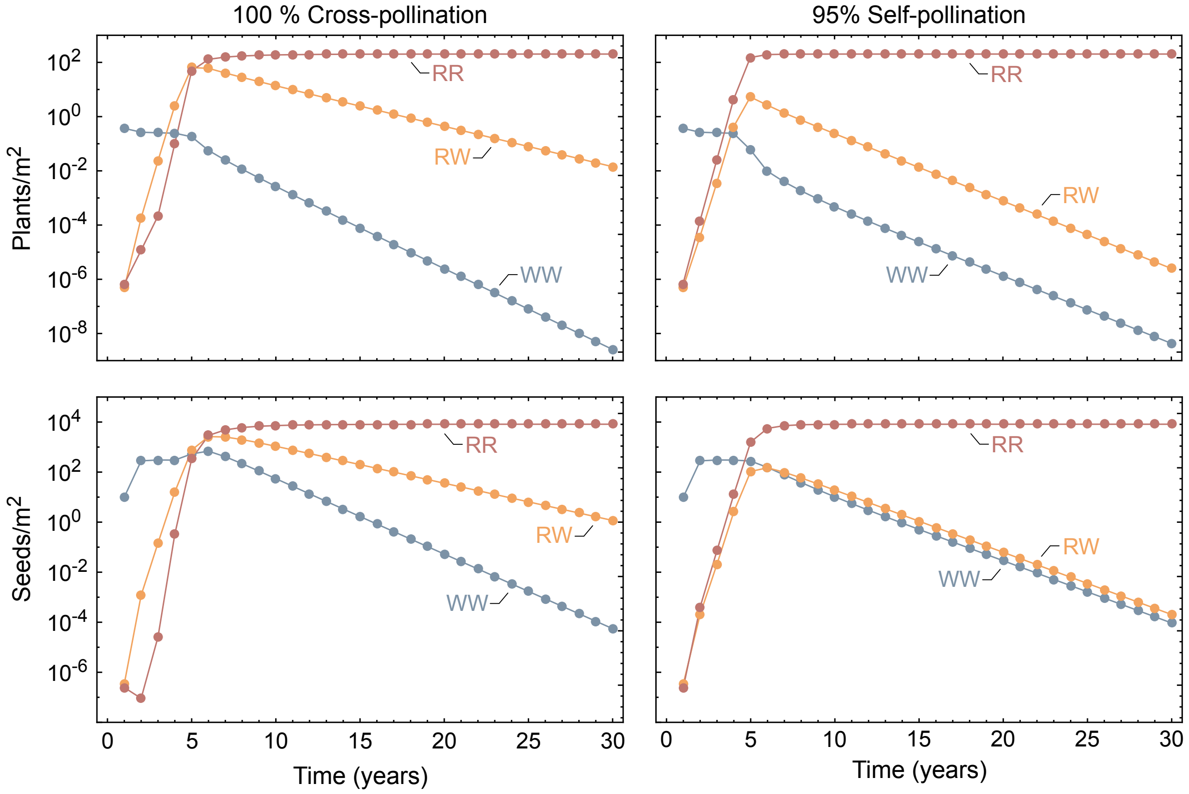

Fig. S2: **Predicted target-site resistance evolution in herbicide-treated Johnsongrass depending on self-pollination.** The results are obtained for a partially dominant resistance allele ($k_h = 0.5$) and fitness cost ($k_c = 0.5$). Shown are predictions of our deterministic model for the genotype composition of Johnsongrass plants ($\widetilde{P}$) and seeds (B) over 30 years of herbicide application for pure cross-pollination and $95\ \%$ self-pollination. The frequency of sensitive plants (WW) is shown in blue, resistant heterozygotes (RW) in yellow and resistant homozygotes (RR) are represented in red.

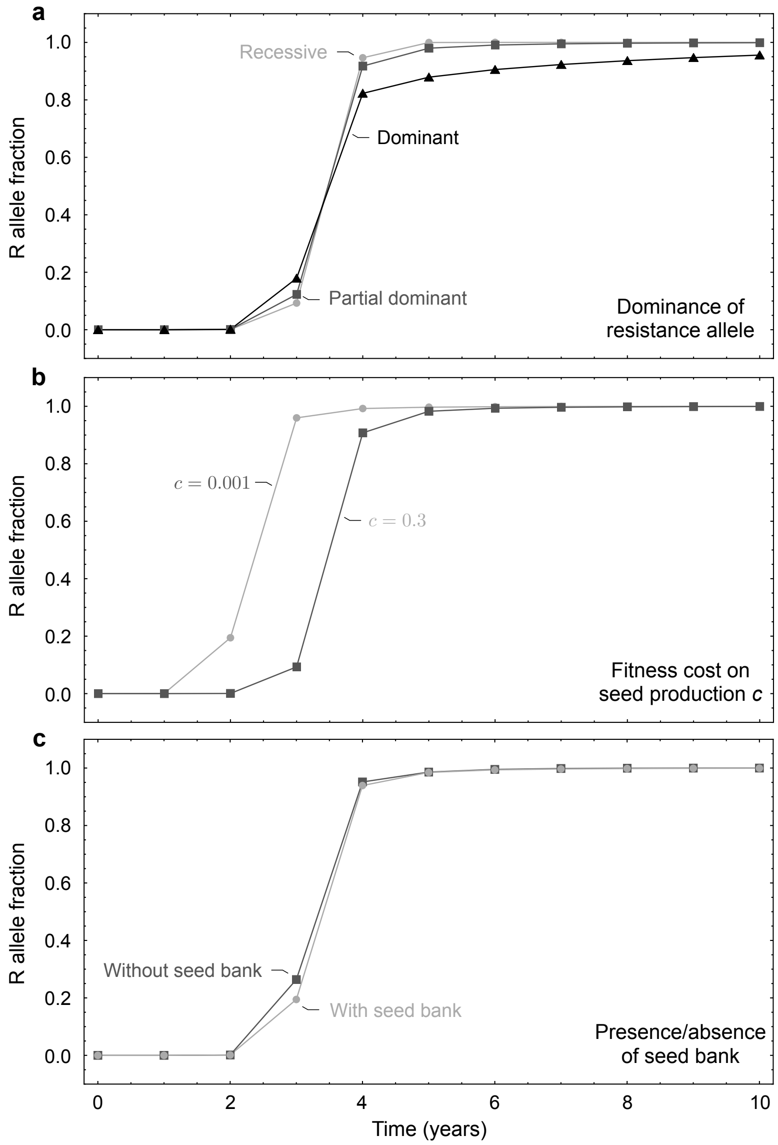

Fig. S3: **Predicted target-site resistance evolution in herbicide-treated Johnsongrass for different degrees of resistance dominance, low and high resistance cost and depending on seed bank formation.** Shown are predictions of our deterministic model obtained for a partially dominant resistance allele ($k_h = 0.5$) (varies in **a**) and fitness cost ($k_c = 0.5$). **a**, Changes in frequency of the resistance allele R in Johnsongrass plants ($\widetilde{P}$) under herbicide application for different degrees of resistance dominance (k_h). Where recessive corresponds to $k_h = 0$ (light grey line with closed circles), partially dominant to $k_h = 0.5$ (dark grey line with closed squares) and dominant to $k_h = 1$ (black line with closed triangles). **b**, Changes in frequency of the resistance allele R in Johnsongrass plants ($\widetilde{P}$) under herbicide application for low ($c = 0.001$) and high ($c = 0.3$) resistance cost. The initial genotype composition differs between the low (light grey line with closed circles) and high (dark grey line with closed squares) fitness cost (compare Fig. 2 b). **c**, Changes in frequency of the resistance allele R in Johnsongrass plants ($\widetilde{P}$) under herbicide application depending on the formation of a seed bank. The light grey line with closed circles corresponds to a population with a seed bank and the dark grey line with closed squares to a population without a seed bank.

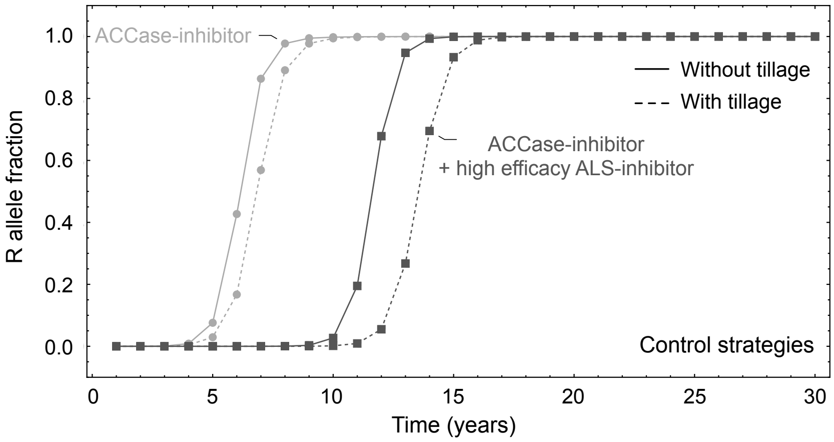

Fig. S4: **Predicted target-site resistance evolution in herbicide-treated Johnsongrass for different control regimes.** Shown are deterministic predictions for the changes in the frequency of the resistance allele R in Johnsongrass plants ($\widetilde{P}$) under different control strategies. The distinct control strategies are: ACCase-inhibitor (light grey line with closed circles) or ACCase-inhibitor and ALS-inhibitor with equal efficacy (dark grey line with closed squares) applied solely (solid line) or combined with tillage (dashed line). These dynamics where obtained with a different version of our population-based model. Compared to the model presented in this manuscript, this model does not include density dependence in reproduction and no mortality of seeds before entering the seed bank. Furthermore, the parameter set is based on the one used by Liu et al. (2019) along with a yearly seed bank mortality of 20 %. The implementation with all parameter values is available on GitHub at `https://github.com/tecoevo/JohnsongrassDynamics`.

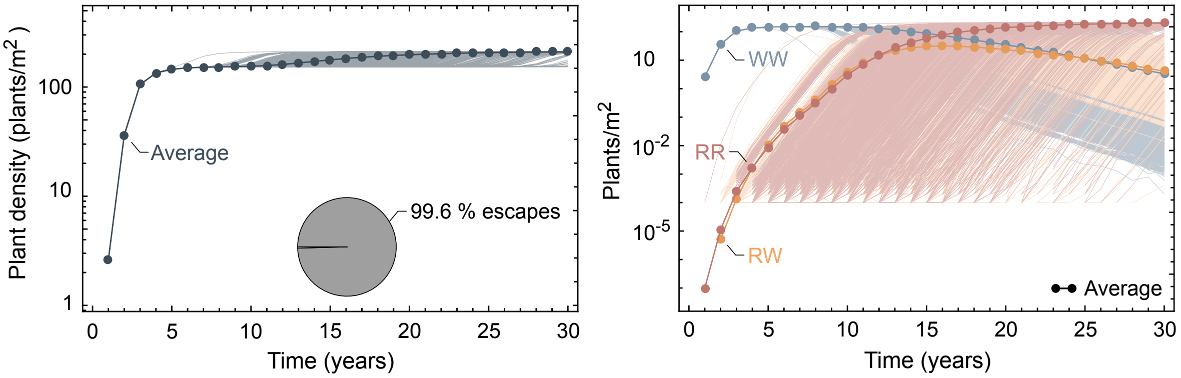

Fig. S5: **Simulated population dynamics and target-site resistance evolution in Johnsongrass treated with low efficacy ALS-inhibitor.** Shown are changes in Johnsongrass density ($\widetilde{P}/A$) and genotype composition of plants ($\widetilde{P}$) over 30 years of ALS-inhibitor application with low efficacy of 1000 simulation runs obtained for a partially dominant resistance allele ($k_h = 0.5$) and fitness cost ($k_c = 0.5$). The thick lines with closed circles correspond to the average of all simulation runs, and the thin lines represent the individual realisations. The frequency of sensitive seeds (WW) is shown in blue, resistant heterozygotes (RW) in yellow and resistant homozygotes (RR) are represented in red. The pie charts display the proportion of simulation runs where the weed population escapes from control and regrowths due to herbicide resistance evolution.

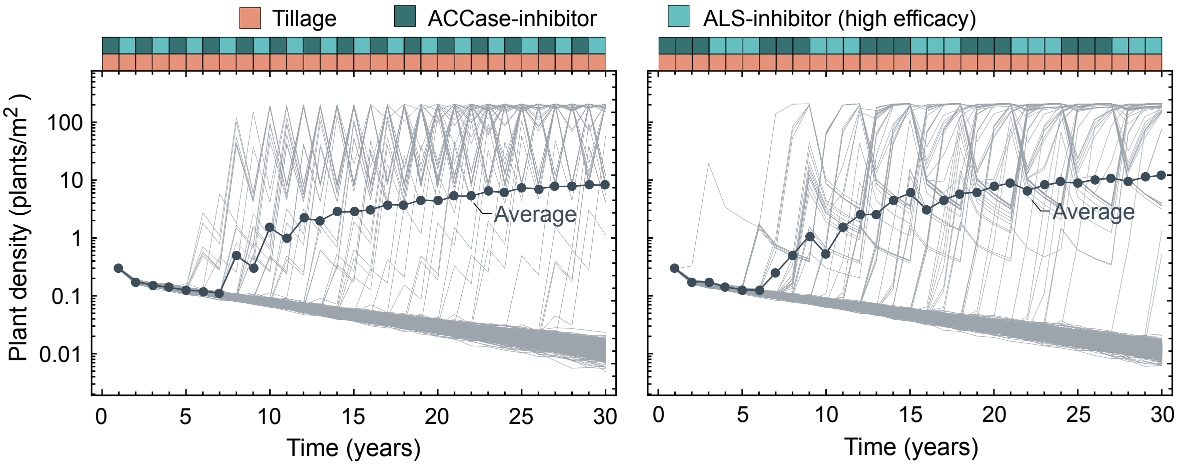

Fig. S6: **Simulated population dynamics in Johnsongrass for annual and three-annual herbicide rotations.** The simulation results are obtained for a partially dominant resistance allele ($k_h = 0.5$) and fitness cost ($k_c = 0.5$). The left panel shows changes in Johnsongrass density ($\widetilde{P}/A$) over 30 years for a yearly rotation of ACCase- and ALS-inhibitor combined with soil tillage. Population dynamics under a rotation of the same herbicides with a cycle length of three years combined with tillage are displayed in the right panel.

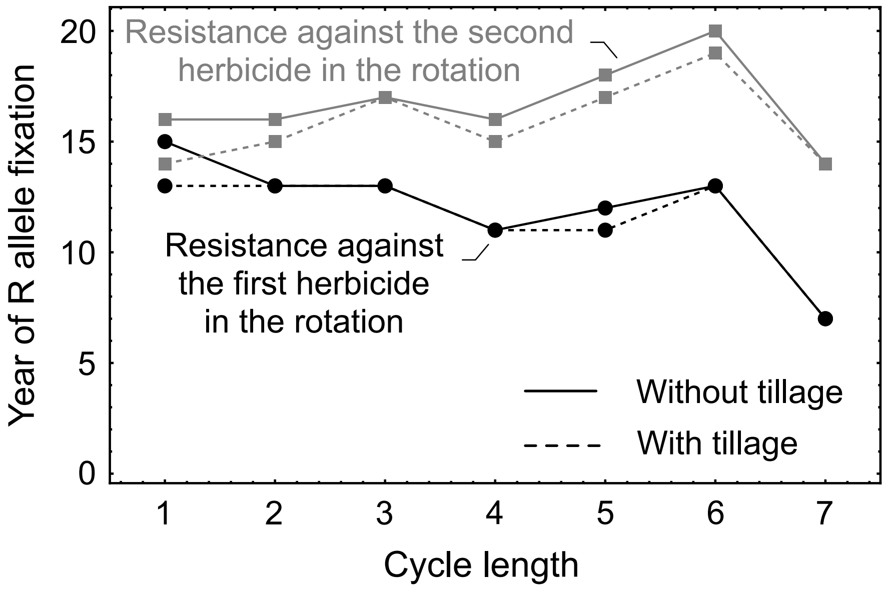

Fig. S7: **Predicted fixation time of target-site resistance in Johnsongrass under binary herbicide rotations.** The results are obtained for a partially dominant resistance allele ($k_h = 0.5$) and fitness cost ($k_c = 0.5$). Considered are rotations of two herbicides with distinct target sites, ACCase- and ALS-inhibitors, Johnsongrass is known to develop target-site resistance towards. We assume equal efficacy here. The cycle length refers to the number of years one herbicide is recurrently applied before the treatment switches to the other herbicide. The year of resistance allele fixation is when the resistance allele frequency in plants ($\widetilde{P}$), obtained from the deterministic model, reaches $99.5\ \%$. The black lines with closed circles show the fixation of target-site resistance against the first herbicide in the rotation, and the grey line with closed rectangles resistance fixation against the second herbicide applied, where the herbicides are applied without mechanical control (solid line) or combined with tillage (dashed line).

References

Acciaresi, H. A. & Chidichimo, H. O. (2005), ‘Ecophysiological response of Sorghum halepense populations to reduced rates of nicosulfuron’, *Pesquisa Agropecuária Brasileira* **40**(6), 541–547.

Anderson, L. E., Appleby, A. P. & Weseloh, J. W. (1960), ‘Characteristics of Johnsongrass Rhizomes’, *Weeds* **8**(3), 402.

Bagavathiannan, M. V. & Norsworthy, J. K. (2013), ‘Postdispersal Loss of Important Arable Weed Seeds in the Midsouthern United States’, *Weed Science* **61**(4), 570–579.

Bargués-Ribera, M. & Gokhale, C. S. (2020), ‘Eco-evolutionary agriculture: Host-pathogen dynamics in crop rotations’, *PLoS Computational Biology* **16**(1), e1007546.

Baucom, R. S. (2019), ‘Evolutionary and ecological insights from herbicide-resistant weeds: what have we learned about plant adaptation, and what is left to uncover?’, *New Phytologist* **223**(1), 68–82.

Beasley, C. A. (1970), ‘Development of Axillary Buds from Johnsongrass Rhizomes’, *Weed Science* **18**(2), 218–222.

Beckie, H. J., Busi, R., Bagavathiannan, M. V. & Martin, S. L. (2019), ‘Herbicide resistance gene flow in weeds: Under-estimated and under-appreciated’, *Agriculture, Ecosystems & Environment* **283**, 106566.

Bescansa, P., Imaz, M., Virto, I., Enrique, A. & Hoogmoed, W. (2006), ‘Soil water retention as affected by tillage and residue management in semiarid spain’, *Soil and Tillage Research* **87**(1), 19–27.

Bliss, C. I. (1939), ‘The Toxicity of Poisons Applied Jointly’, *Annals of Applied Biology* **26**(3), 585–615.

Busi, R., Powles, S. B., Beckie, H. J. & Renton, M. (2020), ‘Rotations and mixtures of soil-applied herbicides delay resistance’, *Pest Management Science* **76**(2), 487–496.

Délye, C., Deulvot, C. & Chauvel, B. (2013), ‘Dna analysis of herbarium specimens of the grass weed alopecurus myosuroides reveals herbicide resistance pre-dated herbicides’, *PLOS ONE* **8**(10).

Diggle, A. J., Neve, P. B. & Smith, F. P. (2003), ‘Herbicides used in combination can reduce the probability of herbicide resistance in finite weed populations’, *Weed Research* **43**(5), 371–382.

Dogan, M. N. & Boz, O. (2005), ‘The concept of reduced herbicide rates for the control of johnsongrass (Sorghum halepense (L.) Pers.) in cotton during the critical period for weed control’, *Journal of Plant Diseases and Protection* **112**, 71–79.

Egley, G. H. & Chandler, J. M. (1978), ‘Germination and Viability of Weed Seeds After 2.5 Years in a 50-Year Buried Seed Study’, *Weed Science* **26**(3), 230–239.

Firbank, L. G. & Watkinson, A. R. (1985), ‘On the Analysis of Competition Within Two-Species Mixtures of Plants’, *The Journal of Applied Ecology* **22**(2), 503.

Freckleton, R. P. & Stephens, P. A. (2009), ‘Predictive models of weed population dynamics’, *Weed Research* **49**(3), 225–232.

Ghersa, C., Satorre, E. & Esso, M. V. (1985), ‘Seasonal Patterns of Johnsongrass Seed Production in Different Agricultural Systems’, *Israel Journal of Plant Sciences* **34**(1), 24–30.

Harms, C. T. & DiMaio, J. J. (1991), ‘Primisulfuron Herbicide-resistant Tobacco Cell Lines. Application of Fluctuation Test Design to in vitro Mutant Selection with Plant Cells’, *Journal of Plant Physiology* **137**(5), 513–519.

Harris, T. E. (1963), *The Theory of Branching Processes*, Springer, Berlin.

Hartzler, R. G., Gover, A. & Stellingwerf, J. (1991), ‘Factors Affecting Winter Survival of Johnsongrass (Sorghum halepense) Rhizomes’, *Weed Technology* **5**(1), 108–110.

Haughn, G. & Somerville, C. R. (1987), *Selection for Herbicide Resistance at the Whole-Plant Level*, American Chemical Society, chapter 7, pp. 98–107.

Hernndez, M. J., Len, R., Fischer, A. J., Gebauer, M., Galdames, R. & Figueroa, R. (2015), ‘Target-Site Resistance to Nicosulfuron in Johnsongrass (Sorghum halepense) from Chilean Corn Fields’, *Weed Science* **63**(3), 631–640.

Holm, L. G., Plucknett, D. L., Pancho, J. V. & Herberger, J. P. (1977), *The World’s Worst Weeds: Distribution and Biology*, East-West Center, University Press of Hawaii.

Holmes, K. H., Lindquist, J. L., Rebarber, R., Werle, R., Yerka, M. & Tenhumberg, B. (2022), ‘Modeling the evolution of herbicide resistance in weed species with a complex life cycle’, *Ecological Applications* **32**(1), e02473.

Holst, N., Rasmussen, I. A. & Bastians, L. (2007), ‘Field weed population dynamics: a review of model approaches and applications’, *Weed Research* **47**(1), 1–14.

Horowitz, M. (1972), ‘Early Development of Johnsongrass’, *Weed Science* **20**(3), 271–273.

Hull, R. J. (1970), ‘Germination Control of Johnsongrass Rhizome Buds’, *Weed Science* **18**(1), 118–121.

Johnson, D. B. & Norsworthy, J. K. (2014), ‘Johnsongrass (Sorghum halepense) Management as Influenced by Herbicide Selection and Application Timing’, *Weed Technology* **28**(1), 142–150.

Johnson, W. G. & Frans, R. E. (1991), ‘Johnsongrass (Sorghum halepense) Control in Soybeans (Glycine max) with Postemergence Herbicides’, *Weed Technology* **5**(1), 87–91.

Johnson, W. G., Li, J. & Wait, J. D. (2003), ‘Johnsongrass Control, Total Nonstructural Carbohydrates in Rhizomes, and Regrowth After Application of Herbicides Used in Herbicide-Resistant Corn (Zea mays)1’, *Weed Technology* **17**(1), 36–41.

Keeley, P. E. & Thullen, R. J. (1979), ‘Influence of Planting Date on the Growth of Johnsongrass (Sorghum halepense) from Seed’, *Weed Science* **27**(5), 554–558.

Klein, P. & Smith, C. M. (2021), ‘Invasive Johnsongrass, a threat to native grasslands and agriculture’, *Biologia* **76**(2), 413–420.

Kreiner, J. M., Stinchcombe, J. R. & Wright, S. I. (2017), ‘Population Genomics of Herbicide Resistance: Adaptation via Evolutionary Rescue’, *Annual Review of Plant Biology* **69**(1), 1–25.

Krenchinski, F., Albrecht, A., Albrecht, L., Villetti, H., Orso, G., Barroso, A. & Victoria, R. (2015), ‘Germination and Dormancy in Seeds of Sorghum halepense and Sorghum arundinaceum’, *Planta Daninha* **33**(2), 223–230.

Landry, R. L., Stephenson, D. O. & Woolam, B. C. (2016), ‘Glufosinate Rate and Timing for Control of Glyphosate-Resistant Rhizomatous Johnsongrass (Sorghum halepense) in Glufosinate-Resistant Soybean’, *International Journal of Agronomy* **2016**, 1–6.

Legutzamón, E. S. (1986), ‘Seed survival and patterns of seedling emergence in Sorghum halepense (L.) Pers.’, *Weed Research* **26**(6), 397–404.

Liu, C., Scursoni, J. A., Moreno, R., Zelaya, I. A., Muñoz, M. S. & Kaundun, S. S. (2019), 'An individual-based model of seed- and rhizome-propagated perennial plant species and sustainable management of Sorghum halepense in soybean production systems in Argentina', *Ecology and Evolution* **9**(17), 10017–10028.

Loddo, D., Masin, R., Otto, S. & Zanin, G. (2012), 'Estimation of base temperature for Sorghum halepense rhizome sprouting', *Weed Research* **52**(1), 42–49.

Lolas, P. C. & Coble, H. D. (1980), 'Johnsongrass (Sorghum halepense) growth characteristics as related to rhizome length', *Weed Research* **20**(4), 205–210.

Lorentz, L. (2014), Herbicide Resistance - Molecular and Physiological Characterization of the Glyphosate Resistant Weeds Amaranthus ssp. and Sorghum ssp., PhD thesis, Rheinische Friedrich-Wilhelms-Universität Bonn.

Maxwell, B. D., Roush, M. L. & Radosevich, S. R. (1990), 'Predicting the Evolution and Dynamics of Herbicide Resistance in Weed Populations', *Weed Technology* **4**(1), 2–13.

McWhorter, C. G. (1961), 'Morphology and Development of Johnsongrass Plants from Seeds and Rhizomes', *Weeds* **9**(4), 558.

McWhorter, C. G. (1971), 'Growth and Development of Johnsongrass Ecotypes', *Weed Science* **19**(2), 141–147.

McWhorter, C. G. & Hartwig, E. E. (1965), 'Effectiveness of Preplanting Tillage in Relation to Herbicides in Controlling Johnsongrass for Soybean Production1', *Agronomy Journal* **57**(4), 385–389.

Menchari, Y., Chauvel, B., Darmency, H. & Délye, C. (2008), 'Fitness costs associated with three mutant acetyl-coenzyme A carboxylase alleles endowing herbicide resistance in black-grass Alopecurus myosuroides', *Journal of Applied Ecology* **45**(3), 939–947.

Ohadi, S., Hodnett, G., Rooney, W. & Bagavathiannan, M. (2018), 'Gene Flow and its Consequences in Sorghum spp.', *Critical Reviews in Plant Sciences* **36**(5-6), 1–19.

Panozzo, S. & Sattin, M. (2021), 'Fitness Costs Associated to an Ile2041Asn Mutation in the Geophyte Sorghum halepense Resistant to ACCase-Inhibiting Herbicides', *Frontiers in Agronomy* **3**, 711840.

Paterson, A. H., Kong, W., Johnston, R. M., Nabukalu, P., Wu, G., Poehlman, W. L., Goff, V. H., Isaacs, K., Lee, T.-H., Guo, H., Zhang, D., Sezen, U. U., Kennedy, M., Bauer, D.,

Feltus, F. A., Weltzien, E., Rattunde, H. F., Barney, J. N., Barry, K., Cox, T. S. & Scanlon, M. J. (2020), 'The Evolution of an Invasive Plant, Sorghum halepense L. ('Johnsongrass')', *Frontiers in Genetics* **11**, 317.

Peerzada, A. M., Ali, H. H., Hanif, Z., Bajwa, A. A., Kebaso, L., Frimpong, D., Iqbal, N., Namubiru, H., Hashim, S., Rasool, G., Manalil, S., Meulen, A. v. d. & Chauhan, B. S. (2017), 'Eco-biology, impact, and management of Sorghum halepense (L.) Pers.', *Biological Invasions* pp. 1–19.

Preston, C. & Powles, S. B. (2002), 'Evolution of herbicide resistance in weeds: initial frequency of target site-based resistance to acetolactate synthase-inhibiting herbicides in Lolium rigidum', *Heredity* **88**(1), 8–13.

Sager, G. R. & Mortimer, A. M. (1976), 'An approach to the study of the population dynamics of plants with special reference to weeds', *Applied Biology* **1**, 1–47.

Scarabel, L., Panozzo, S., Savoia, W. & Sattin, M. (2014), 'Target-Site ACCase-Resistant Johnsongrass (Sorghum halepense) Selected in Summer Dicot Crops', *Weed Technology* **28**(2), 307–315.

Schwinning, S., Meckel, H., Reichmann, L. G., Polley, H. W. & Fay, P. A. (2017), 'Accelerated development in Johnsongrass seedlings (Sorghum halepense) suppresses the growth of native grasses through size-asymmetric competition', *PLOS ONE* **12**(5), e0176042.

Scopel, A. L., Ballare, C. L. & Ghersa, C. M. (1988), 'Role of Seed Reproduction in the Population Ecology of Sorghum halepense in Maize Crops', *The Journal of Applied Ecology* **25**(3), 951.

Skøien, S. E., Børresen, T. & Bechmann, M. (2012), 'Effect of tillage methods on soil erosion in norway', *Acta Agriculturae Scandinavica, Section B — Soil & Plant Science* **62**(sup2), 191–198.

Stoller, E. (1977), 'Differential Cold Tolerance of Quackgrass and Johnsongrass Rhizomes', *Weed Science* **25**(4), 348–351.

Torra, J., Montull, J. M., Taberner, A., Onkokesung, N., Boonham, N. & Edwards, R. (2021), 'Target-Site and Non-target-Site Resistance Mechanisms Confer Multiple and Cross- Resistance to ALS and ACCase Inhibiting Herbicides in Lolium rigidum From Spain', *Frontiers in Plant Science* **12**, 625138.

Tóth, V. & Lehoczky, E. (2006), ‘Investigations on the germination depth of Johnson grass (Sorghum halepense [L.] pers)’, *Communications in Agricultural and Applied Biological Sciences* **71**(3), 803—808.

Tuesca, D., Puricelli, E., Nisensohn, L., Faccini, D. & Papa, J. (1999), ‘Decision criteria for optimizing postemergence johnsongrass control in soybean crops in Argentina’, *Pesquisa Agropecuária Brasileira* **34**(5), 749–753.

Vidrine, P. R. (1989), ‘Johnsongrass (Sorghum halepense) Control in Soybeans (Glycine max) with Postemergence Herbicides’, *Weed Technology* **3**(3), 455–458.

Vila-Aiub, M. M., Balbi, M. C., Gundel, P. E., Ghersa, C. M. & Powles, S. B. (2007), ‘Evolution of Glyphosate-Resistant Johnsongrass (Sorghum halepense) in Glyphosate-Resistant Soybean’, *Weed Science* **55**(6), 566–571.

Vrbničanin, S., Božić, D. & Pavlović, D. (2017), Gene flow from herbicide-resistant crops to wild relatives, *in* Z. Pacanoski, ed., ‘Herbicide Resistance in Weeds and Crops’, IntechOpen, Rijeka, chapter 3.

Warwick, S. I. & Black, L. (1983), ‘The biology of Canadian weeds. 61. Sorghum halepense (L.) Pers.’, *Canadian Journal of Plant Science* **63**, 997–1014.

Warwick, S. I., Phillips, D. & Andrews, C. (1986), ‘Rhizome depth: the critical factor in winter survival of Sorghum halepense (L.) Pers. (Johnson grass)’, *Weed Research* **26**(6), 381–388.

Watkinson, A. (1980), ‘Density-dependence in single-species populations of plants’, *Journal of Theoretical Biology* **83**(2), 345–357.

Weisberger, D., Nichols, V. & Liebman, M. (2019), ‘Does diversifying crop rotations suppress weeds? A meta-analysis’, *PLoS ONE* **14**(7), e0219847.

Williams, R. & Ingber, B. (1977), ‘The Effect of Intraspecific Competition on the Growth and Development of Johnsongrass under Greenhouse Conditions’, *Weed Science* **25**(4), 293–297.

Winton-Daniels, K., Frans, R. & McClelland, M. (1990), ‘Herbicide Systems for Johnsongrass (Sorghum halepense) Control in Soybeans (Glycine max)’, *Weed Technology* **4**(1), 115–122.

Yoda, K., Kira, T., Ogawa, H. & Hozumi, K. (1963), ‘Self-Thinning in Overcrowded Pure Stands under Cultivated and Natural Conditions’, *Journal of Biology* **14**, 107–129.

Zeller, A. K., Zeller, Y. I. & Gerhards, R. (2021), ‘A long-term study of crop rotations, herbicide strategies and tillage practices: Effects on Alopecurus myosuroides Huds. Abundance and contribution margins of the cropping systems’, *Crop Protection* **145**, 105613.
