# Supplementary material for: Theoretical assessment of persistence and adaptation in weeds with complex life cycles
Source: Nat Plants. 2023 Aug 3;9(8):1267–79. doi: 10.1038/s41477-023-01482-1 (PMC10435386; doi:10.1038/s41477-023-01482-1)
Supplement: Supplementary file 2 — Reporting Summary [file 41477_2023_1482_MOESM2_ESM.pdf]

nature portfolio

Corresponding author(s): Dana Lauenroth

Last updated by author(s): Sep 14, 2022

Reporting Summary

Nature Portfolio wishes to improve the reproducibility of the work that we publish. This form provides structure for consistency and transparency in reporting. For further information on Nature Portfolio policies, see our Editorial Policies and the Editorial Policy Checklist.

Statistics

For all statistical analyses, confirm that the following items are present in the figure legend, table legend, main text, or Methods section.

n/a	Confirmed	
☒	☐	The exact sample size (*n*) for each experimental group/condition, given as a discrete number and unit of measurement
☒	☐	A statement on whether measurements were taken from distinct samples or whether the same sample was measured repeatedly
☒	☐	The statistical test(s) used AND whether they are one- or two-sided<br>*Only common tests should be described solely by name; describe more complex techniques in the Methods section.*
☒	☐	A description of all covariates tested
☒	☐	A description of any assumptions or corrections, such as tests of normality and adjustment for multiple comparisons
☐	☒	A full description of the statistical parameters including central tendency (e.g. means) or other basic estimates (e.g. regression coefficient) AND variation (e.g. standard deviation) or associated estimates of uncertainty (e.g. confidence intervals)
☒	☐	For null hypothesis testing, the test statistic (e.g. *F*, *t*, *r*) with confidence intervals, effect sizes, degrees of freedom and *P* value noted<br>*Give P values as exact values whenever suitable.*
☒	☐	For Bayesian analysis, information on the choice of priors and Markov chain Monte Carlo settings
☒	☐	For hierarchical and complex designs, identification of the appropriate level for tests and full reporting of outcomes
☒	☐	Estimates of effect sizes (e.g. Cohen's *d*, Pearson's *r*), indicating how they were calculated

Our web collection on statistics for biologists contains articles on many of the points above.

Software and code

Policy information about availability of computer code

Data collection	The population-based model was parameterized based on published data as stated in the supporting information. The deterministic version of the model as well as stochastic simulation, based on binomial, Poisson and multinomial distributed random numbers, were implemented in MATLAB_R2021b. To generate the binomial random numbers an implementation of the bnldev algorithm (Numerical Recipes in Fortan 90 p. 155-156) in MATLAB_R2021b was used. All data used in the study was generated with the custom codes available on GitHub.
Data analysis	The data was generated with custom made code implemented in MATLAB_R2021b and figures were created with custom code implemented in Mathematica 13.2.0.0. All the custom codes are available on GitHub at https://github.com/tecoevo/JohnsongrassDynamics

For manuscripts utilizing custom algorithms or software that are central to the research but not yet described in published literature, software must be made available to editors and reviewers. We strongly encourage code deposition in a community repository (e.g. GitHub). See the Nature Portfolio guidelines for submitting code & software for further information.

Data

Policy information about availability of data

All manuscripts must include a data availability statement. This statement should provide the following information, where applicable:

- Accession codes, unique identifiers, or web links for publicly available datasets
- A description of any restrictions on data availability
- For clinical datasets or third party data, please ensure that the statement adheres to our policy

The generated data is available on GitHub at https://github.com/tecoevo/JohnsongrassDynamics

Human research participants

Policy information about studies involving human research participants and Sex and Gender in Research.

Reporting on sex and gender	No human research participants were included in our study.
Population characteristics	No human research participants were included in our study.
Recruitment	No human research participants were included in our study.
Ethics oversight	No human research participants were included in our study.

Note that full information on the approval of the study protocol must also be provided in the manuscript.

Field-specific reporting

Please select the one below that is the best fit for your research. If you are not sure, read the appropriate sections before making your selection.

☐ Life sciences ☐ Behavioural & social sciences ☒ Ecological, evolutionary & environmental sciences

For a reference copy of the document with all sections, see nature.com/documents/nr-reporting-summary-flat.pdf

Ecological, evolutionary & environmental sciences study design

All studies must disclose on these points even when the disclosure is negative.

Study description	A population- based model in discrete time was developed to describe the population dynamics and herbicide resistance evolution in perennial weed species like Sorghum halepense. The model was parameterized based on published data as stated in the supporting information. The deterministic version of the model as well as stochastic simulation, based on binomial, Poisson and multinomial distributed random numbers, were implemented in Matlab. Data generated with this codes was used to analyse the population dynamics and resistance evolution under different genetic assumptions and control regimes.
Research sample	There were no research samples collected for the study. The model was parameterized based on published data as stated in the supporting information.
Sampling strategy	There were no research samples collected for the study.
Data collection	The data was generated via computer simulations implemented in Matlab, by Dana Lauenroth, and automatically saved in txt files in csv format.
Timing and spatial scale	There were no research samples collected for the study.
Data exclusions	The data was created via computer simulations, no research samples were collected for the study and therefore no data had to be excluded.
Reproducibility	All code used to create the data and the generated data sets are published on GitHub.
Randomization	Randomization was not necessary as there were no research samples collected for the study.
Blinding	Blinding was neither possible nor necessary as the data was created via computer simulations.

Did the study involve field work? ☐ Yes ☒ No

Reporting for specific materials, systems and methods

We require information from authors about some types of materials, experimental systems and methods used in many studies. Here, indicate whether each material, system or method listed is relevant to your study. If you are not sure if a list item applies to your research, read the appropriate section before selecting a response.

Materials & experimental systems

n/a	Involved in the study
☒	☐ Antibodies
☒	☐ Eukaryotic cell lines
☒	☐ Palaeontology and archaeology
☒	☐ Animals and other organisms
☒	☐ Clinical data
☒	☐ Dual use research of concern

Methods

n/a	Involved in the study
☒	☐ ChIP-seq
☒	☐ Flow cytometry
☒	☐ MRI-based neuroimaging
